# Unveiling the ion sensing capabailities of ‘click’ derived chalcone-tailored 1,2,3-triazolic isomers for Pb(ii) and Cu(ii) ions: DFT analysis†

**DOI:** 10.1039/d4ra01471e

**Published:** 2024-05-13

**Authors:** Riddima Singh, Gurleen Singh, Nancy George, Gurjaspreet Singh, Pooja Malik, Harminder Singh, Gurpreet Kaur, Jandeep Singh

**Affiliations:** a School of Chemical Engineering and Physical Sciences, Lovely Professional University Phagwara–144411 Punjab India singhjandeep@gmail.com; b Department of Chemistry and Centre of Advanced Studies in Chemistry, Panjab University Chandigarh–160014 India; c Department of Chemistry, Gujranwala Guru Nanak Khalsa College, Civil Lines Ludhiana–141001 Punjab India

## Abstract

In this study, two novel chalcone-derived 1,2,3-triazole-appended positional isomers (probe 6 and probe 9) were synthesized *via* the ‘CuAAC’ (Cu(i) – catalysed alkyne azide cycloaddition) methodology for the purpose of metal ion detection. The synthesized probes underwent characterization utilizing standard spectroscopic methodologies including FTIR, NMR (^1^H and ^13^C), and mass spectrometry. Subsequently, the sensing capabilities of these probes were explored using UV-Vis and fluorescence spectroscopy, wherein their selective recognition potential was established for Pb(ii) and Cu(ii), both of which can pose serious health hazards when prevalent in the environment above permissible limits. Both the probes exhibited fairly low limits of detection (LoD), determined as 5.69 μM and 6.55 μM in the case of probe 6 for Pb(ii) and Cu(ii) respectively; whereas the probe 9 exhibited an LoD of 5.06 μM and 7.52 μM for Pb(ii) and Cu(ii), respectively. The job's plot for the probe demonstrates the formation of a 1 : 1 complex between the metal and ligand. Furthermore, the interaction of the free probes with the metal ions in the metal–ligand complex was elucidated through ^1^H NMR analysis and validated theoretically using Density Functional Theory (DFT) simulations with the B3LYP/6-311G++(d,p) and B3LYP/LANL2DZ basis sets for geometry optimization of the probes and their corresponding metal complexes. These findings offer a reliable approach to Cu(ii) and Pb(ii) ion detection and can be further used for the potential applications in environmental monitoring and analytical chemistry.

## Abstract 2

Two new chalcone-based 1,2,3-triazole derivatives have been synthesised using ‘CuAAC’ for Cu(ii) and Pb(ii) ion detection.

## Graphical abstract

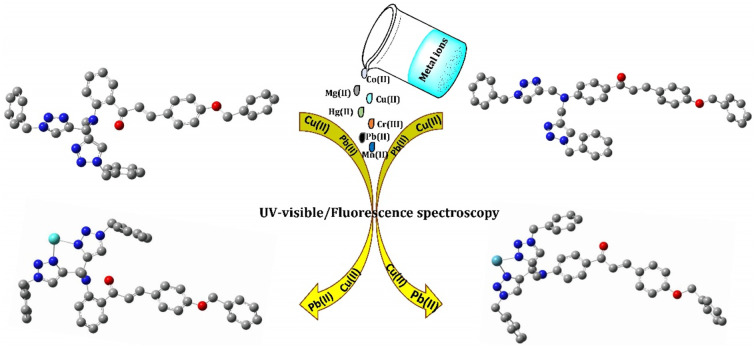

## 1. Introduction

The toxic heavy metal ions, such as copper (Cu) and lead (Pb), pose significant ecological concerns due to their persistence in the environment and the capacity to accumulate within various ecosystems.^1,2^ These metal ions are primarily released into the environment through anthropogenic activities such as industrial processes, mining, and improper waste disposal.^3^ Once introduced, they undergo complex biogeochemical cycles and can accumulate in soils, sediments, and aquatic systems, thereby proving challenging as they tend to biomagnify through the food chain, leading to higher concentrations in organisms at higher trophic levels.^4,5^ The adverse effects of copper and lead accumulation in living organisms are manifold. Copper, an essential micronutrient at low concentrations, becomes toxic when present in excess, leading to oxidative stress, cellular damage, and disruption of enzyme functions.^6^ On the other hand, Lead is a non-essential metal but can still interfere with calcium metabolism, disrupt nerve transmission, impair reproductive and developmental processes, and even at low levels, pose severe neurological risks, especially to children.^7^ The ecological and physiological impacts of copper and lead accumulation underscore the critical importance of sensing and monitoring these toxic heavy metal ions in the environment to preserve the health of ecosystems and safeguard living organisms.^8^ For this purpose, 1,2,3-triazole-based chemosensors, synthesized *via* ‘click chemistry’ provide a reliable alternative that is simple to implement and yields accurate findings with high selectivity and sensitivity.^9–11^

Click chemistry constitutes a methodological paradigm within chemical synthesis, characterized by its expeditious and high-yield generation of products.^12^ Among its diverse modalities, the Copper(i)-catalyzed Azide–Alkyne Cycloaddition (CuAAC) has emerged as a preeminent avenue for constructing 1,2,3-triazoles.^13^ This transformative reaction involves the cyclization of an azide and alkyne moiety, mediated by a copper(i) catalyst, culminating in the regioselective and efficient formation of the 1,2,3-triazole scaffold. The versatility of CuAAC in producing 1,2,3-triazoles has found remarkable applications, particularly in the development of selective ion sensors.^14–16^ By judiciously designing triazole-based ligands, these sensors can be tailored to detect specific metal ions with high sensitivity and selectivity. As a result, the coordination abilities of the 1,2,3-triazole moiety enable it to interact selectively with certain metal ions, leading to distinct changes in the photophysical properties.^17^ This technological synergy has been harnessed to effectually identify an array of metal ions, encompassing copper, lead, mercury, and zinc, within the purview of environmental, biomedical, and analytical purviews.^18,19^ The application of 1,2,3-triazoles as selective chemosensors, underscores its significance in constructing functional materials with ion sensing capabilities, contributing to advancements in both chemical synthesis and analytical sciences.

For synthetic chemists, using CuAAC to design selective 1,2,3-triazole-based chemosensors can be beneficial due to the method's selectivity, simplicity of experimental setup, tolerance of a broad range of functional groups, and high quantitative yields.^20^ Traditional analytical methods for sensing metal ions, such as atomic absorption spectrometry (AAS)^21^ and inductively coupled plasma mass spectrometry (ICPMS)^22^ are used for sensing metal ions, however, despite their high sensitivity, they are not suitable for on-site investigation owing to their costly instrumentation, extensive set-up time in specialized laboratories, and strenuous sample preparation.^23^

In this investigation, two novel chalcone-based 1,2,3-triazole-appended positional isomeric chemosensors have been synthesized. The design of chalcone-based 1,2,3-triazole ligands aims to enhance conjugation within the system, resulting in altered photophysical properties. Chalcone derivatives, known primarily for absorbing UV light and possessing inherent conjugated systems and strategic functional groups, exhibit fluorescence^24–27^ and are customized for sensitive and selective detection of metal ions. CuAAC methodology was implemented for synthesizing 1,2,3-triazole linked probes 6 and 9 that was characterized by IR, NMR, and mass spectrometry. UV-Vis and fluorescence spectroscopy demonstrate selective binding of the probes to Pb(ii) and Cu(ii) ions, evidenced by significant changes in absorption/emission spectra. Furthermore, DFT studies aid in geometry optimization and molecular electrostatic potential analysis, supporting experimental findings of metal–ligand complexation as validated by ^1^H NMR analysis.

## 2. Experiment

Caution! Sodium azide is heat and shock-sensitive. It requires extreme safety and cautionary measures to handle.

### 2.1. Materials and method

The experiments were conducted under standard laboratory conditions. The chloride salts of Ca(ii), Co(ii), Ba(ii), Cr(iii), Hg(ii), Mg(ii), Cu(ii), Ni(ii), Pb(ii), Ce(iii), Na(i), k(i), Mn(ii) and Zn(ii) were procured from LOBA. The initial compound, 4-benzyloxybenzaldehyde, was obtained from spectrochem. Additionally, 2-amino acetophenone (LOBA), 4-amino acetophenone (LOBA), anhydrous potassium carbonate (LOBA), tetrahydrofuran (THF) (LOBA), ethanol, benzyl chloride (CDH), triethylamine (Et_3_N) (SDFCL), sodium azide (LOBA), [CuBr(PPh_3_)_3_] (Sigma Aldrich), and *N*,*N*-dimethylformamide (DMF) (LOBA), propargyl bromide (80% by weight in toluene) (Spectrochem) were sourced from various suppliers as indicated. Benzyl azide was synthesized *via* a known procedure by reacting benzyl chloride with dried sodium azide.^28^ Spectroscopic analyses were performed using a SHIMADZU FTIR-8400S Spectrometer and a BRUKER-ADVANCE-II FT-NMR-AL 500 MHz spectrometer, with chemical shifts in NMR referenced against tetramethylsilane. Mass spectrometry (LCMS) was carried out using a Bruker Esquire 300 mass spectrometer, and melting points were determined using a Labtronics LT 108 device. Elemental analyses (CHN) were conducted using a PerkinElmer Model 2400 CHNS elemental analyzer. Chemosensing analysis employed a SHIMADZU UV-1900 Spectrometer. For DFT analysis, Gaussian software utilizing a hybrid density functional (B3LYP)/631G+(d,p) basis set and LANL2DZ basis set was utilized.

### 2.2. Synthesis of chalcone 4 and 7

**Scheme 1:**
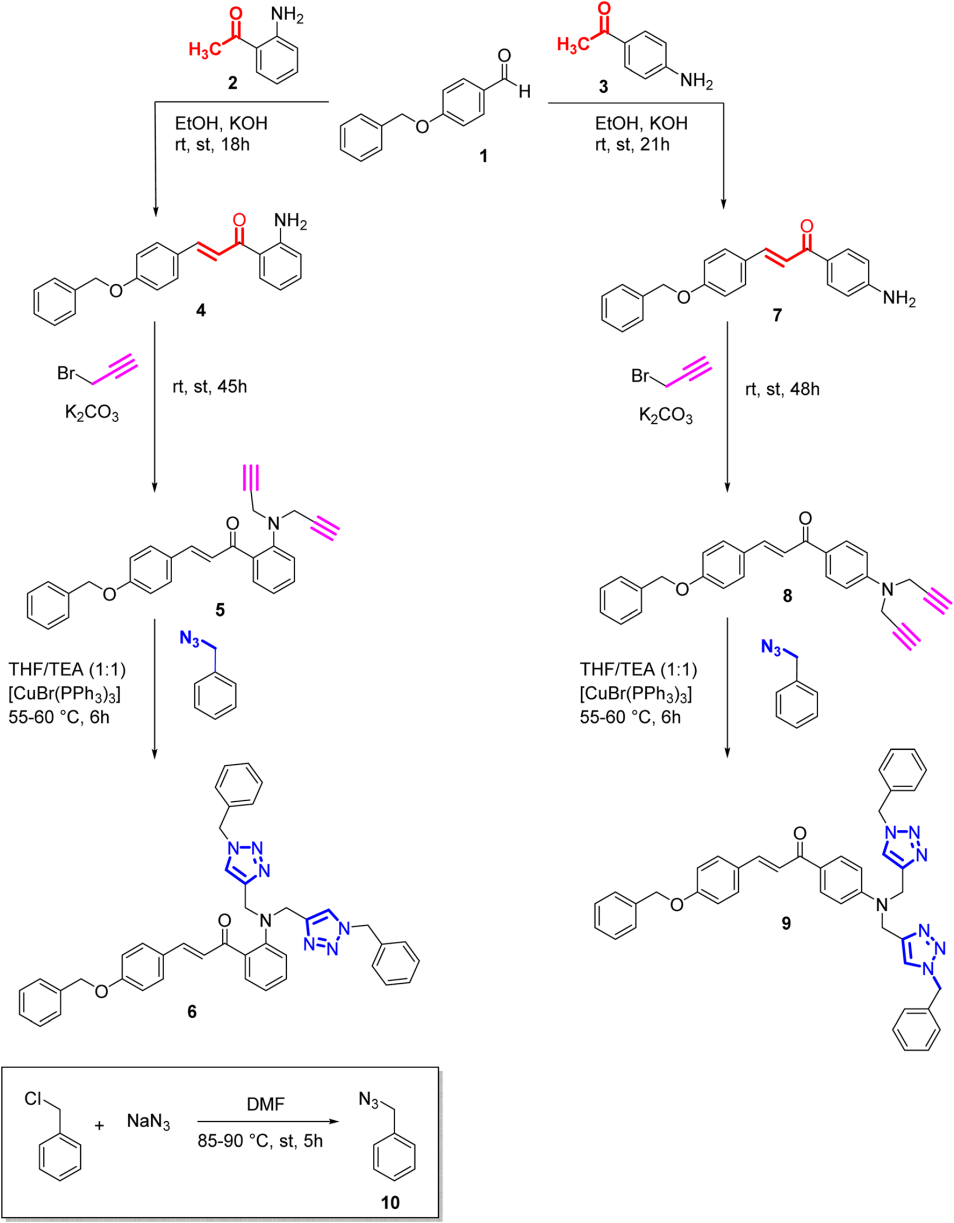
Synthesis of alkynes (5,8) from chalcones and their corresponding triazoles (6,9) *via* 3 + 2 cycloaddition reaction.

The starting reactants were dissolved in ethanol under constant stirring. Potassium hydroxide was gradually introduced into the reaction mixture (Scheme 1). Stirring of the reaction mixture was maintained at room temperature until full conversion of reactants to the intended products, with the progression monitored *via* TLC (ethyl acetate : hexane 1 : 9). Quenching of the reaction was achieved by adding ice-cold water to the reaction mixture, followed by filtration and drying of the solid product. Subsequently, the obtained product was purified using ethanol as the eluent.^20,29^

#### 2.2.1. (*E*)-1-(2-Aminophenyl)-3-(4-(benzyloxy)phenyl)prop-2-en-1-one

The procedure for synthesis as mentioned above was used. The quantities used were: 4-benzyloxybenzaldehyde (1) (2.0 g, 9.4 mmol), 2-aminoacetophenone (1.25 g, 9.4 mmol) (2), potassium hydroxide (5.0 mL, 20% w/v). Yield: 91%; colour: bright yellow; texture: solid powder; M.F. = C_22_H_19_NO_2_; Elem. Anal. Calc. (%): *C* = 80.22; *H* = 5.81; *N* = 4.25; Found (%): *C* = 80.27, *H* = 5.85, *N* = 4.34; Mp.: 135–136 °C; IR (neat, cm^−1^): 3436, 3328, 3044, 1638, 1581, 1502, 1336, 1263, 1205, 1161, 1003, 828, 738, 680, 604, 508.

#### 2.2.2. (*E*)-1-(4-Aminophenyl)-3-(4-(benzyloxy)phenyl)prop-2-en-1-one

The procedure for synthesis as mentioned above was used. The quantities used were: 4-benzyloxybenzaldehyde (1) (2.0 g, 9.4 mmol), 4-aminoacetophenone (1.25 g, 9.4 mmol) (3), potassium hydroxide (5.0 mL, 20% w/v). Yield: 90%; colour: dark yellow; texture: solid powder; M.F. = C_22_H_19_NO_2_; Elem. Anal. Calc. (%): *C* = 80.22; *H* = 5.81; *N* = 4.25; Found (%): *C* = 80.23, *H* = 5.83, *N* = 4.27; Mp.: 119–120 °C; IR (neat, cm^−1^): 3373, 3336, 3192, 3035, 2912, 1624, 1593, 1505, 1454, 1421, 1382, 1341, 1291, 1247, 1220, 1166, 1078, 1017, 986, 911, 817, 733, 692, 629, 594, 514.

### 2.3. Synthesis of alkyne 5 and 8

The chalcone was dissolved in DMF by constantly swirling the solution on a stirrer. Subsequently, anhydrous potassium carbonate was introduced into the solution, followed by the slow addition of propargyl bromide dropwise. The reaction mixture was stirred at ambient temperature, and the reaction was monitored *via* TLC using a solvent mixture of ethyl acetate and hexane. Subsequently, the reaction was quenched by the addition of ice-cold water to the mixture. The resulting solid product was filtered and dried at room temperature.

#### 2.3.1. (*E*)-3-(4-(Benzyloxy)phenyl)-1-(2-(di(prop-2-yn-1-yl)amino)phenyl)prop-2-en-1-one

The procedure for synthesis as mentioned above was used. The quantities used were: chalcone (4) (1.0 g, 3.03 mmol), DMF (20 mL), propargyl bromide (0.78 g, 6.88 mmol), anhydrous potassium carbonate (2.1 g, 15.2 mmol). Yield: 83%; colour: brown; texture: oil; M.F. = C_28_H_23_NO_2_; IR (neat, cm^−1^): 3287, 3029, 2910, 2113, 1637, 1570, 1504, 1456, 1420, 1293, 1245, 1203, 1161, 1077, 1007, 916, 821, 738, 650, 613, 513; ^1^H NMR (500 MHz, CDCl_3_): *δ* = 7.91 (*d*, *J* = 6.7 Hz, 1H), 7.53–7.46 (m, 2H), 7.44–7.30 (m, 8H), 6.98 (*d*, *J* = 8.9 Hz, 4H), 5.08 (s, 2H), 4.02 (s, 4H), 2.23 (s, 2H); ^13^C NMR (126 MHz, CDCl_3_): *δ* = 191.86, 160.53, 150.44, 142.80, 136.52, 134.73, 134.11, 131.50, 130.01, 128.68, 128.17, 127.50, 120.91, 119.49, 115.34, 115.28, 112.04, 80.33, 71.31, 70.12, 32.35.

#### 2.3.2. (*E*)-3-(4-(Benzyloxy)phenyl)-1-(4-(di(prop-2-yn-1-yl)amino)phenyl)prop-2-en-1-one

The procedure for synthesis as mentioned above was used. The quantities used were: chalcone (7) (1.0 g, 3.03 mmol), DMF (20 mL), propargyl bromide (0.78 g, 6.88 mmol), anhydrous potassium carbonate (2.1 g, 15.2 mmol). Yield: 85%; colour: brown; texture: oil; M.F. = C_28_H_23_NO_2_; IR (neat, cm^−1^): 3286, 2970, 2913, 2115, 1651, 1587, 1505, 1421, 1331, 1293, 1220, 1165, 1017, 813, 734, 687, 645, 600, 511; ^1^H NMR (500 MHz, CDCl_3_) *δ* = 7.82 (*d*, *J* = 8.8 Hz, 1H), 7.70 (*d*, *J* = 6.1 Hz, 2H), 7.45–7.37 (m, 6H), 7.06 (*d*, *J* = 8.8 Hz, 2H), 6.82 (*d*, *J* = 7.2 Hz, 2H), 6.68 (*d*, *J* = 8.6 Hz, 2H), 5.08 (s, 2H), 3.98 (*d*, *J* = 2.6 Hz, 4H), 2.25 (*t*, *J* = 2.5 Hz, 2H); ^13^C NMR (126 MHz, CDCl_3_): *δ* = 190.79, 160.46, 150.70, 142.87, 136.50, 131.99, 130.79, 129.97, 128.65, 128.13, 127.47, 121.92, 119.82, 115.36, 115.29, 115.21, 115.15, 112.32, 79.89, 71.86, 70.08, 33.06.

### 2.4. General procedure for the synthesis of benzyl azide (10)

A solution comprising benzyl chloride (5.5 g, 47.8 mmol) in 25 mL of DMF was prepared using a magnetic stirrer, after which sodium azide (15.5 g, 239 mmol) was added. The reaction mixture was then heated to 85–90 °C and refluxed for 4–5 hours (Scheme 1). The successful completion of the reaction was confirmed through TLC analysis, utilizing ethyl acetate and hexane (in a ratio of 1 : 4). The product was extracted utilizing ethyl acetate as the solvent. Following extraction, the combined organic layers were isolated, desiccated with anhydrous sodium sulfate, filtered, and subjected to vacuum evaporation to eliminate any remaining solvent. Yield = 60%; color: light yellow; texture = oil; M.F.: C_7_H_7_N_3_; IR (neat, cm^−1^): 3032, 2930, 2089, 1452, 1252, 876, 697, 568; ^1^H NMR (500 MHz, CDCl_3_) *δ* = 7.27–7.12 (m, 5H), 4.14 (s, 2H) ppm; ^13^C NMR (126 MHz, CDCl_3_) *δ* = 135.53, 128.91, 128.37, 128.31, 54.82 ppm.

### 2.5. Synthesis and characterization of chalcone-based triazole derivatives 6 and 9

The chalcone-based alkyne was dissolved in THF : TEA (3 : 2) was combined with organic azide and Cu(i) (0.001 mmol) was used as a catalyst. The resulting mixture was allowed to reflux for 6 hours at 55–60 °C until complete conversion of reactants into the desired product, monitored by TLC (ethyl acetate : hexane 1 : 4). The reaction was terminated by adding ice-cold water to the mixture, followed by filtration and drying of the solid product.

#### 2.5.1. (*E*)-3-(4-(Benzyloxy)phenyl)-1-(2-(bis((1-benzyl-1*H*-1,2,3-triazol-4-yl)methyl)amino)phenyl)prop-2-en-1-one

The procedure for synthesis as mentioned above was used. The quantities used were: alkyne 5 (0.70 g, 1.73 mmol), THF : TEA (3 : 2), the organic azide 10 (0.46 g, 3.46 mmol). Yield: 93%; colour/texture: yellow powder; M.F.: C_42_H_37_N_7_O_2_; Elem. Anal. Calc. (%): *C* = 75.09; *H* = 5.55; *N* = 14.59, Found (%): *C* = 75.11; *H* = 5.50; *N* = 14.57, Mp.: 162–163 °C; IR (neat, cm^−1^): 3068, 3033, 1638, 1570, 1507, 1455, 1419, 1280, 1242, 1198, 1165, 1009, 823, 736, 701, 607, 516; ^1^H NMR (500 MHz, CDCl_3_): *δ* = 7.91 (*dd*, *J* = 8.1, 1.6 Hz, 2H), 7.84 (*d*, *J* = 16.5 Hz, 1H), 7.69 (*d*, *J* = 15.5 Hz, 2H), 7.57 (*d*, *J* = 8.8 Hz, 3H), 7.41 (*m*, *J* = 11.7, 7.2 Hz, 9H), 7.36–7.33 (m, 6H), 7.00 (*d*, *J* = 8.9 Hz, 2H), 6.80 (*d*, *J* = 8.3 Hz, 2H), 5.47 (s, 4H), 5.10 (s, 2H), 4.60 (*d*, *J* = 5.6 Hz, 4H); ^13^C NMR (126 MHz, CDCl_3_): *δ* = 190.79, 159.49, 150.06, 141.64, 135.45, 133.84, 133.64, 130.46, 128.94, 128.04, 127.63, 127.17, 127.12, 126.92, 126.45, 119.83, 117.97, 114.23, 113.89, 111.10, 69.06, 53.12, 38.04. LC-MS: *m*/*z* (calculated) = 671.81; *m*/*z* (found) = 672.25 (*M*+1).

#### 2.5.2. (*E*)-3-(4-(Benzyloxy)phenyl)-1-(4-(bis((1-benzyl-1*H*-1,2,3-triazol-4-yl)methyl)amino)phenyl)prop-2-en-1-one

The procedure for synthesis as mentioned above was used. The quantities used were: alkyne 8 (0.70 g, 1.73 mmol), THF : TEA (3 : 2), the organic azide 10 (0.46 g, 3.46 mmol). Yield: 86%; colour/texture: dark brown oil; M.F.: C_42_H_37_N_7_O_2_; IR (neat, cm^−1^): 3031, 2958, 2922, 1649, 1590, 1503, 1455, 1293, 1327, 1224, 1169, 1114, 1017, 809, 731, 694, 598, 513; ^1^H NMR (500 MHz, CDCl_3_): *δ* 7.84 (s, 1H), 7.61 (s, 2H), 7.54 (s, 2H), 7.44 (*d*, *J* = 3.3 Hz, 5H), 7.41–7.39 (m, 5H), 7.35 (*dd*, *J* = 6.7, 3.7 Hz, 6H), 7.07 (*d*, *J* = 8.7 Hz, 2H), 6.82 (*d*, *J* = 9.2 Hz, 2H), 6.68 (*d*, *J* = 9.6 Hz, 2H), 5.47 (*d*, *J* = 2.3 Hz, 4H), 5.09 (s, 2H), 4.47 (*d*, *J* = 3.0 Hz, 4H); ^13^C NMR (126 MHz, CDCl_3_): *δ* = 189.79, 166.75, 159.43, 141.71, 135.48, 130.97, 129.87, 128.94, 128.10, 127.77, 127.70, 127.63, 127.29, 127.11, 127.00, 126.45, 118.79, 114.19, 110.98, 69.06, 67.14, 37.70; LC-MS: *m*/*z* (calculated) = 671.81; *m*/*z* (found) = 672.32 (*M*+1).

## 3. Results and discussions

### 3.1. Synthesis

The synthesis of probes 6 and 9 follows a three-step procedure, beginning with the synthesis of chalcone. Initially, the chalcone is synthesized, followed by the conversion of the chalcone into an alkyne through nucleophilic substitution. Potassium carbonate serves as the catalyst to react with the propargyl group, leading to the substitution of dynamic protons of the chalcone with propargyl moieties and yielding a terminal alkyne as the final product. In the subsequent step, the fusion of azide and alkyne takes place, facilitated by tetrahydrofuran (THF) as the solvent, triethylamine (TEA) as the base, and [CuBr(PPh_3_)_3_] as the catalyst, ultimately resulting in the formation of the desired end product. The final stage of the synthesis adheres to the principles of Green Chemistry, emphasizing achieving a complete atom economy.

### 3.2. Spectroscopic analysis

#### 3.2.1. Infrared (IR) spectroscopy

The IR spectroscopy observations for chalcones (4, 7), terminal alkynes (5, 8), benzyl azide (10), and the chalcone-based 1,2,3-triazole derivatives (6, 9) in the range of 4000–500 cm^−1^ agreed with the predicted values. The IR spectrum of terminal alkyne 5 exhibited stretching peaks attributed to C≡C–H and C≡C moieties at 3287 cm^−1^ and 2113 cm^−1^, respectively, while terminal alkyne 8 displayed similar peaks at 3286 cm^−1^ and 2115 cm^−1^, confirming successful alkyne synthesis. In the IR spectrum of benzyl azide, a prominent peak at 2089 cm^−1^ was observed, corresponding to the N_3_ stretching of the azide group. However, the IR spectra of both 1,2,3-triazole derivatives 6 and 9 lacked peaks at 3287 cm^−1^ or 2113 cm^−1^, and 3286 cm^−1^ or 2115 cm^−1^, respectively, as well as at 2089 cm^−1^, indicating the cycloaddition of the alkyne and azide moieties into the 1,2,3-triazole ring.

#### 3.2.2. NMR spectroscopy and mass spectrometry

The NMR (^1^H and ^13^C) analysis further supported the successful formation of the terminal alkynes (5, 8) and the 1,2,3-triazole derivatives (6, 9). The peak at *δ* = 2.23 ppm in the ^1^H NMR spectrum of the terminal alkyne 5 and peaks at *δ* = 70.12 ppm and *δ* = 80.33 ppm in ^13^C NMR spectrum, was absent in the ^1^H NMR and ^13^C NMR spectrum of the 1,2,3-triazole derivative 6. The protons of the 1,2,3-triazole ring were attributed to the peak at *δ* = 7.69 ppm. The peak at *δ* = 71.31 ppm was attributed to the methylene C atom, was shifted downfield to *δ* = 69.06 ppm. Similarly, the ^1^H NMR spectrum of alkyne 8 displayed a peak at *δ* = 2.25 ppm and peaks at *δ* = 71.86 ppm and *δ* = 79.89 ppm in ^13^C NMR spectrum was absent in ^1^H NMR and ^13^C NMR spectrum of the 1,2,3-triazole derivative 9. The protons of the 1,2,3-triazole ring were attributed to the peak at *δ* = 7.61 ppm. The peak at *δ* = 70.08 ppm was attributed to the methylene C atom was shifted downfield to *δ* = 69.06 ppm, thereby confirming the conversion of the alkyne moieties into the 1,2,3-triazole derivatives. The mass spectra of both the 1,2,3-triazole derivatives 6 and 9 exhibited peaks at *m*/*z* = 671.81 (theoretical *m*/*z* = 672.25 *M* + 1)), and *m*/*z* = 671.81 (theoretical *m*/*z* = 672.32 (M + 1), respectively.

### 3.3. UV-Vis analysis to scrutinize chemosensing behavior

**Fig. 1:**
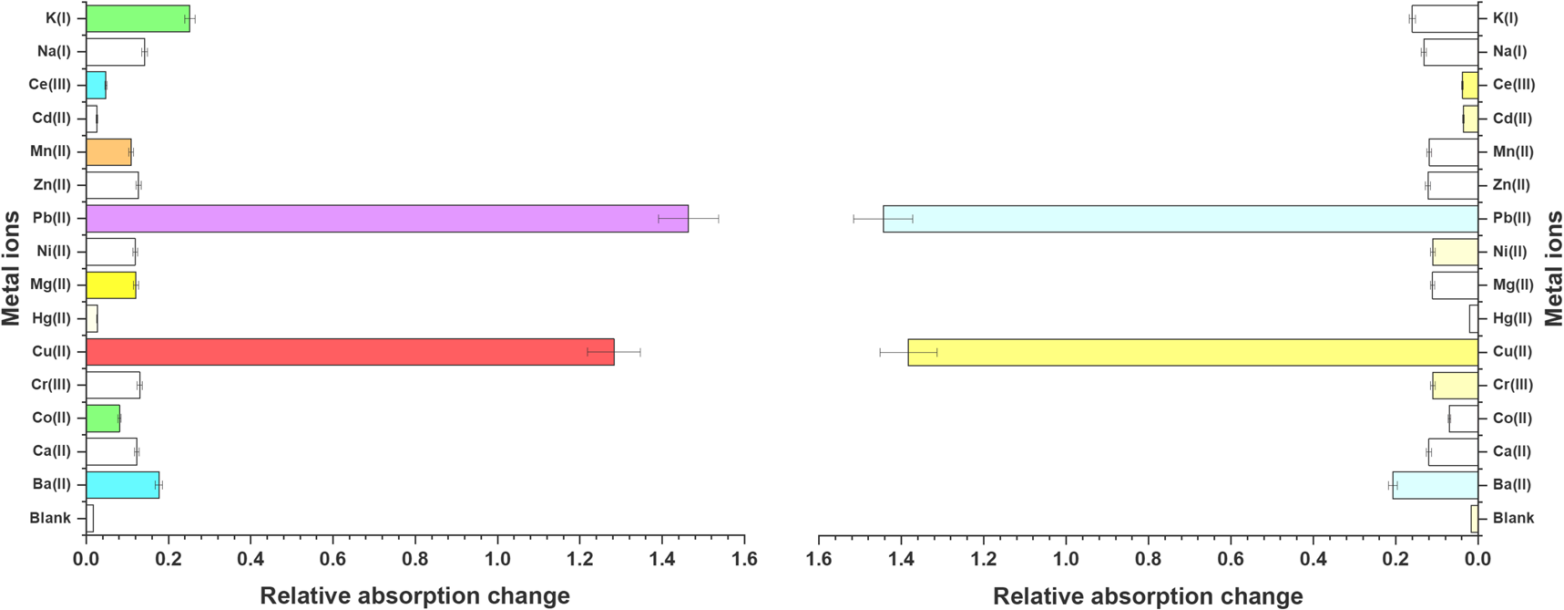
Graphical representation of relative absorption changes of probes 6 and 9 on addition of 15 equivalents of various metal ions.

The UV-Vis spectral investigations for evaluating the chemosensing behavior of probes 6 and 9 yielded substantial results to establish their ion recognition potential. Since both the probes were soluble in DMSO, it was used as the solvent for the UV-Vis spectroscopy experiments. The optimized concentrations of 0.5 and 0.03 mM for the solution of probes 6 and 9 respectively, were selected for monitoring the chemosensing response due to the adequate absorption bands. The absorption peak of probe 6 was observed at 340 nm, while probe 9 was observed at 368 nm. The chlorides of Ba(ii), Ca(ii), Co(ii), Cr(iii), Cu(ii), Hg(ii), Mg(ii), Ni(ii), Pb(ii), Zn(ii), Mn(ii), Cd(ii), Ce(iii), Na(i), and K(i) were dissolved in DMSO and solutions were prepared, then utilized to evaluate the sensitivity of the probes to these ions. Except for Pb(ii) and Cu(ii), which displayed significant shifts in absorbance peaks upon titration with the probe solutions, no notable changes in absorption peaks and/or intensity were observed for other metal ions. Fig. 1 illustrates the relative chemosensing behavior of probes 6 and 9 towards various metal ions at equimolar concentrations, indicating a notably higher response of both probes to Pb(ii) and Cu(ii) compared to other metal ions. In spite of the change in the position of the benzoyl unit, both probe 6 and probe 9 exhibit consistent sensing capabilities towards the target metal ions, indicating that their ability to interact effectively with the analytes remains unaltered. This is attributed to the overall molecular framework and same binding cavity of both the probes (N atoms of triazole ring), which facilitate successful interactions with the target species. However, discernible differences are evident in the limit of detection, limit of quantification, and binding constant values between the two probes.

#### 3.3.1. Response of probe 6 towards Pb(ii) and Cu(ii)

**Fig. 2:**
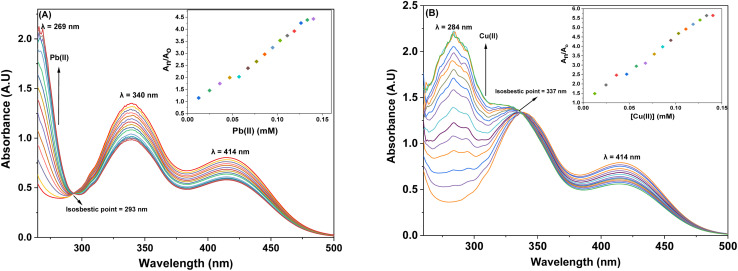
The absorption spectrum of probe 6 upon incremental addition of 15 equivalents of 1 mM metal ions (A) Pb(ii) and (B) Cu(ii); insets illustrate the relative change in absorbance plotted against metal ion concentration (mM).

**Fig. 3:**
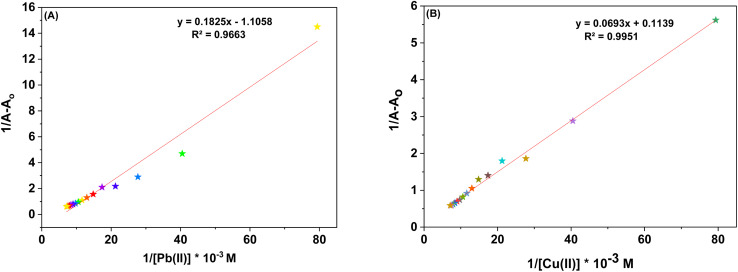
(A) B–H plot for the metal complexation with probe 6 with Pb(ii) (B) B–H plot for the metal complexation with probe 6 with Cu(ii).

The UV-Vis spectral analysis of probe 6 was conducted by titrating a 0.5 mM solution of the probe with separate 1 mM solutions of Pb(ii) and Cu(ii). The resulting spectra are illustrated in Fig. 2(A) and (B) respectively. When Pb(ii) ions were incrementally added, the emergence of absorption peaks at 340 nm and 414 nm due to the n → π* interaction, underwent a hypochromic shift, while the emergence of peak at 269 nm due to π → π* interaction^24^ exhibited an intense hyperchromic shift, thereby resulting in the emergence of an isosbestic point at 293 nm. In the case of Cu(ii) ions, their successive addition to the probe solution led to a hypochromic shift at 414 nm, the peak at 340 nm was barely affected. However, a new intense broad peak emerged at 284 nm and the formation of isosbestic point at 337 nm, thereby confirming the sensing of the metal ions by the probe. The relative change in the absorbance of the probe for Pb(ii) and Cu(ii) is plotted in the insets of Fig. 2(A) and (B), wherein *A*_n_ = absorbance maxima with sequential addition of the metal ions, and *A*_o_ = absorbance maxima of probe 6. Furthermore, the association constant (*K*_a_) was determined from the Benesi–Hildebrand eqn (1) and came out to be 6.18 × 10^3^ M^−1^ and 7.57 × 10^3^ M^−1^. Also, the corresponding B–H plots for Pb(ii) and Cu(ii) have been presented in Fig. 3(A) and (B) respectively. Additionally, the Job plot revealed that probe 6 had a metal-to-ligand binding ratio of 1 : 1 for both Pb(ii) and Cu(ii) (Fig. S28 and S29†) respectively.1
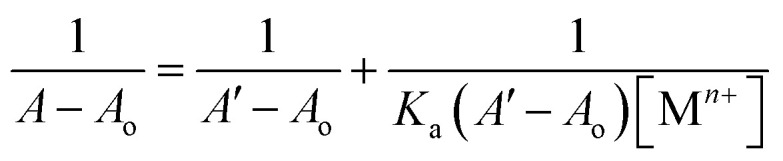
Here, *A*_o_ denotes the initial absorbance intensity, *A* represents the absorbance intensity with a specific concentration of metal ions, *A*′ signifies the final absorbance intensity, [M^*n*+^] indicates the concentration of metal ions, and *K*_a_ stands for the association constant.

#### 3.3.2. Response of probe 9 towards Pb(ii) and Cu(ii)

**Fig. 4:**
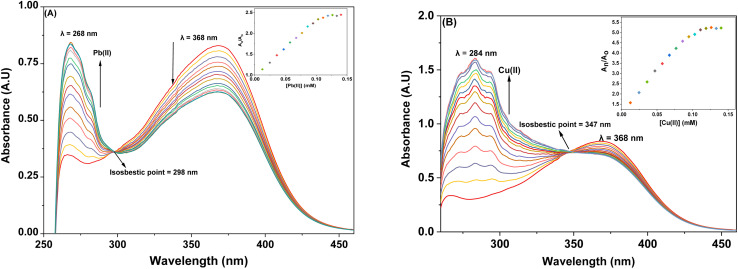
The observed absorption spectrum of probe 9 with the successive addition of 15 equiv. of 1 mM metal ions (A) Pb(ii) (B) Cu(ii); insets display the relative change in the absorbance plotted against metal ion concentration (mM).

**Fig. 5:**
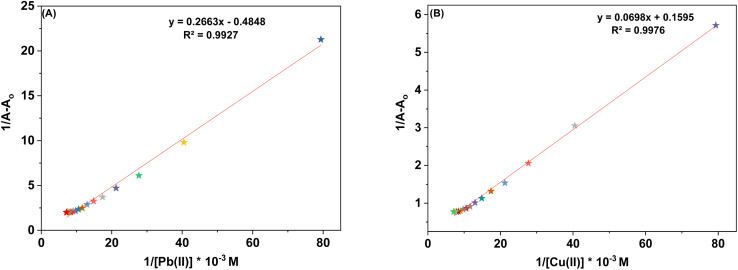
(A) B–H plot for the metal complexation with probe 9 with Pb(ii) (B) B–H plot for the metal complexation with probe 9 with Cu(ii).

The spectral response of probe 9, *via* UV-Vis spectroscopy for Pb(ii) and Cu(ii) ions has been illustrated in Fig. 4(A) and (B) respectively. As can be seen, the incremental addition of Pb(ii) ions to the probe 9 solution resulted in a hypochromic shift at the peak emerged at 368 nm due to n → π* interaction, and a simultaneous intense hyperchromic shift at the peak emerged at 268 nm due to π → π* interaction, thereby exhibiting a ratiometric change, while displaying an isosbestic point at 298 nm. In the case of titration with Cu(ii) ions, the peak at 368 nm witnessed a hypochromic response, however, the peak at 284 nm displayed an intense hyperchromic shift accompanied by a hypsochromic shift of about 16 nm, resulting in a peak at 284 nm. The isosbestic point emerged at 347 nm, confirming the sensing of Cu(ii) ions by the probe 9. The ratiometric response of the probe 9 accompanied by a blue shift is attributable to intramolecular charge transfer (ICT)^17^ in case of binding with both the metal ions (the same holds good for probe 6 also). The relative change in the absorbance of the probe for Pb(ii) and Cu(ii) is plotted in the insets of Fig. 4(A) and (B), wherein *A*_n_ = absorbance maxima with sequential addition of the metal ions, and *A*_o_ = absorbance maxima of probe 9. The association constants for the same were determined from the B–H eqn (1) and came out to be 8.54 × 10^3^ M^−1^ and 9.37 × 10^3^ M^−1^, and the corresponding B–H plots were presented in Fig. 5(A) and (B) respectively. Additionally, the Job plot revealed that probes 9 had a metal-to-ligand binding ratio of 1 : 1 for both Pb(ii) and Cu(ii) (Fig. S30 and S31†) respectively.

#### 3.3.3. Time-dependent studies for probes 6 and 9

The study investigated the influence of time on the interaction between probes 6 and 9 with the Pb(ii) and Cu(ii) ions over 20 minutes. The findings indicated that both probes exhibited consistent behavior with time when interacting with either of the metal ions. The spectral data revealed a constant binding intensity for the probes, evident from a graph (Fig. S20–S23†) that remained a straight line running parallel to the *x*-axis. Additionally, it's noteworthy that the probes' absorption intensity changed rapidly and instantly upon the introduction of either of these ions into the probe solution.

#### 3.3.4. Temperature-dependent analysis for probes 6 and 9

The binding efficiency of probes for the temperature change has been explored by recording absorption spectra of solutions containing probes 6 and 9 complexed with Cu(ii) and Pb(ii) at 2 °C intervals, starting from room temperature and extending up to 50 °C. The observations (Fig. S24–S27†) showed that the interaction between the metal ions and the probes exhibited a minor sensitivity to temperature, as evidenced by the slight increase in absorption intensity with rising temperature.

#### 3.3.5. Competitive metal ion titrations for probes 6 and 9

**Fig. 6:**
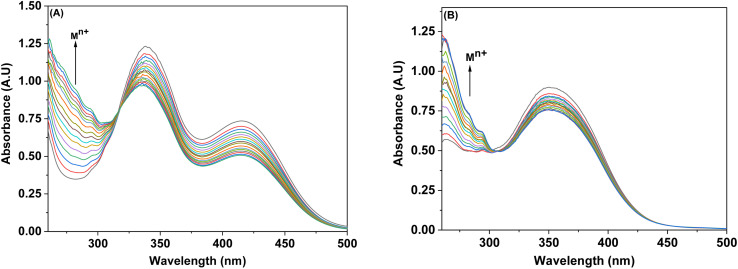
Competitive metal ion titration graph depicting the selective binding of Pb(ii) in (A) probe 6 (B) probe 9.

The imperative trait of numerous potential metal ion sensors lies in their capacity to selectively sense specific metal ions amidst other metal ions. Through titration involving competitive ions, it was determined that probes 6 and 9 possess the capability to selectively identify Pb(ii) in the presence of diverse metal ions. The competitive metal ion titration process involved the incremental addition of a solution containing equimolar concentrations of various metal ions (in DMSO). Analysis of the absorption spectrum after titration revealed that the probe's (6 and 9) tendency to specifically sense Pb(ii) ion remained unaltered despite the presence of other metal ions, as represented in Fig. 6(A) and (B). Notably, the absorption intensity in response to the equimolar concentration solution exhibited a slight reduction when compared to that observed with pure Pb(ii).

### 3.4. Fluorescence studies

#### 3.4.1. Ion sensing behavior of probe 6 and 9 with Pb(ii) and Cu(ii) ions *via* fluorescence spectroscopy

**Fig. 7:**
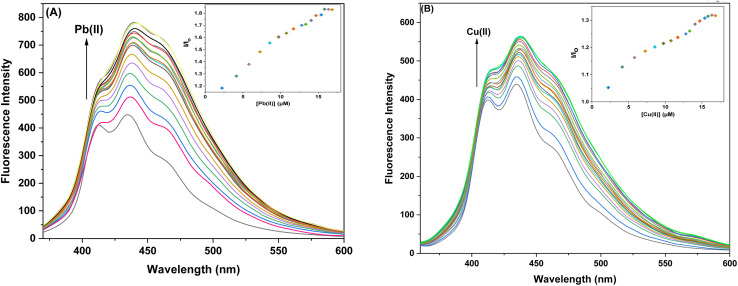
Emission spectra of probe 6 (A) Exhibiting enhanced intensity at different titrations with Pb(ii); insets display the relative change in the intensity (*I*/*I*_o_) plotted against metal ion concentration (μM) (B) Exhibiting enhanced intensity at different titations with Cu(ii); insets display the relative change in the intensity (*I*/*I*_o_) plotted against metal ion concentration (μM).

**Fig. 8:**
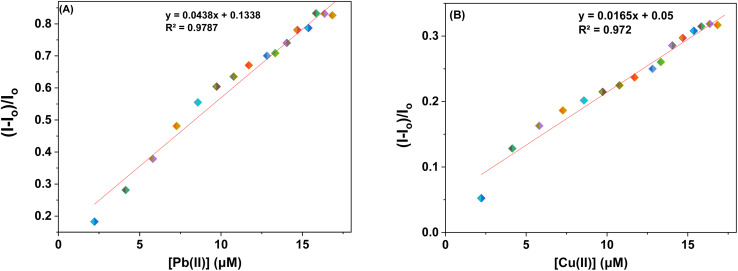
Correlation plots depicting the relative emission intensity (*I* − *I*_o_)/*I*_o_*versus* the molar concentration of (a) Pb(ii) and (b) Cu(ii) ions based on the emission spectra of probe 6. *I*_o_ = primary fluorescence emission of probe 6, while *I* = fluorescence emission of probe 6 in the presence of specific metal ion concentrations (μM).

**Fig. 9:**
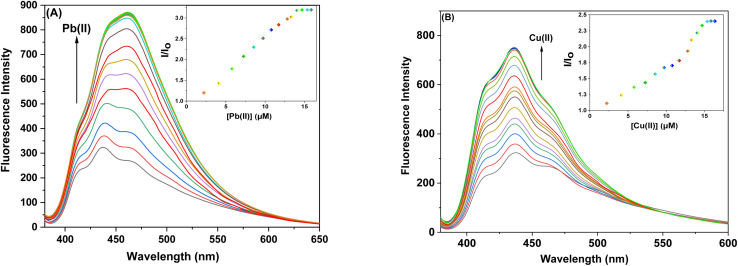
Emission spectra of probe 9 (A) Exhibiting enhanced intensity at different titrations with Pb(ii); insets display the relative change in the intensity (*I*/*I*_o_) plotted against metal ion concentration (μM) (B) Exhibiting enhanced intensity at different titrations with Cu(ii); insets display the relative change in the intensity (*I*/*I*_o_) plotted against metal ion concentration (μM).

**Fig. 10:**
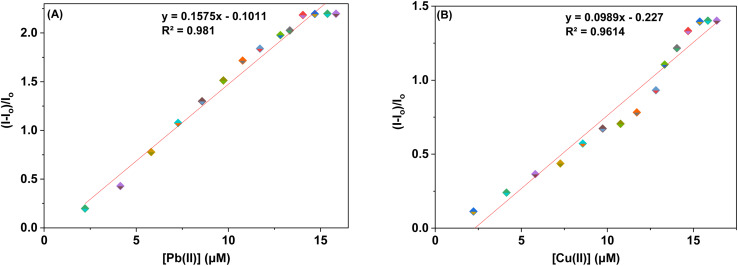
Correlation plots depicting the relative emission intensity (*I* − *I*_o_)/*I*_o_*versus* the molar concentration of (a) Pb(ii) and (b) Cu(ii) ions based on the emission spectra of probe 9. *I*_o_ = primary fluorescence emission of probe 9, while *I* = fluorescence emission of probe 9 in the presence of specific metal ion concentrations (μM).

The sensor probes 6 and 9 were also analyzed for complexation with the Pb(ii) and Cu(ii) ions *via* fluorescence spectroscopy. Upon exposure to an excitation wavelength (*λ*_ex_) of 350 nm, it was noted that probe 6 exhibited a prominent emission peak (*λ*_em_) at 438 nm, along with a minor shoulder peak at 412 nm for both Pb(ii) and Cu(ii) ions, as depicted in Fig. 7(A) and (B). Similarly, when an excitation wavelength (*λ*_ex_) of 370 nm is supplied for probe 9, a highly pronounced emission peak (*λ*_em_) at 436 nm is observed, accompanied by a minor shoulder peak at 412 nm, for both Cu(ii) and Pb(ii), as shown in Fig. 9(A) and (B). Upon conducting independent titrations, it was observed that the fluorescence emission peaks of both probes 6 and 9 exhibited increased intensity upon the introduction of metal ions into the solution containing the probes. The concentration of the solution was consistently maintained at 30 μM throughout the experimental procedure. The concentration range of the metal ions exhibited an incremental increase, reaching up to 15 equivalents. The inset of each emission spectrum demonstrates the proportional change in emission intensity (*I*/*I*_o_) relative to the concentration of metal ions, where *I* represent the fluorescence emission intensity of the probe with the addition of metal ions, and *I*_o_ represents the fluorescence intensity of the probe in the absence of metal ions. In addition, analysis of the correlation plot (*I*_o_ − *I*_n_)/*I*_o_*versus* Pb(ii) concentration and correlation plot (*I*_o_ − *I*_n_)/*I*_o_*versus* Cu(ii) concentration indicated that the probe 6 has a detection limit of 5.69 μM for Pb(ii) and 6.55 μM for Cu(ii). The correlation plot showed that probe 9 had a limit of detection (LoD) of 5.06 μM for Pb(ii) and 7.52 μM for Cu(ii). Additionally, the correlation plots of probe 6 and probe 9 for Pb(ii) and Cu(ii) (Fig. 8 and 10, respectively) were utilized to determine the limit of detection (LoD), the limit of quantification (LoQ), and the binding constant (values have been compiled in Table 1). Additionally, the data presented in the Table 2 provides a comparative analysis of the Limit of Detection (LoD) of chemosensors synthesized previously and the current studies. The probes in this study has been reported for the detection of Pb(ii) and Cu(ii) ions. To the best of our knowledge, the limit of detection for Cu(ii) is comparably low of these sensors as compared to other reported chalcone based sensors (Table 2, entry 8, 9, 10).

**Table 1:** LoD, LoQ, association constant, and stoichiometry of probes 6 and 9 on interaction with the respective metal ion

Probe	Metal ion	LoD (μM)	LoQ (μM)	Association constant (*K*_a_) (M^−1^)	Stoichiometry
Probe 6	Pb(ii)	5.69	18.97	6.18 × 10^3^	1 : 1
	Cu(ii)	6.55	21.85	7.57 × 10^3^	
Probe 9	Pb(ii)	5.06	16.89	8.54 × 10^3^	1 : 1
	Cu(ii)	7.52	25.08	9.37 × 10^3^	

**Table 2:** Previously reported triazole-based chemosensors for the detection of Cu(ii) ions

Entry	Chemosensor	Structure	Limit of detection for copper(ii) (μM)	Reference
1	Triazole-linked glucofuranose derivative	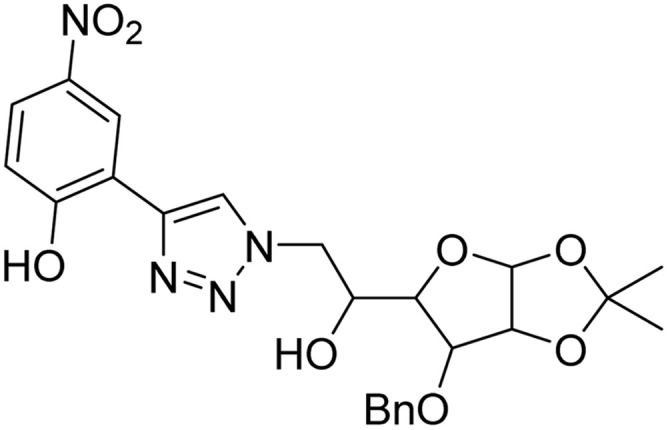	3.50	30
2	Ferrocene–triazole derivative	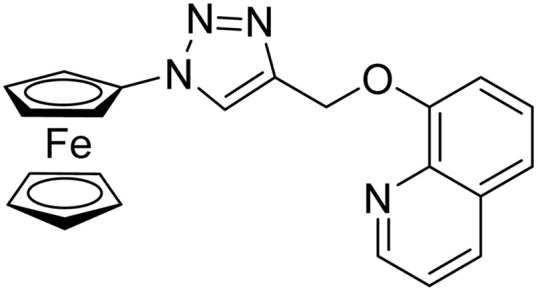	1.71	31
3	Coumarin-based tripodal chemosensor	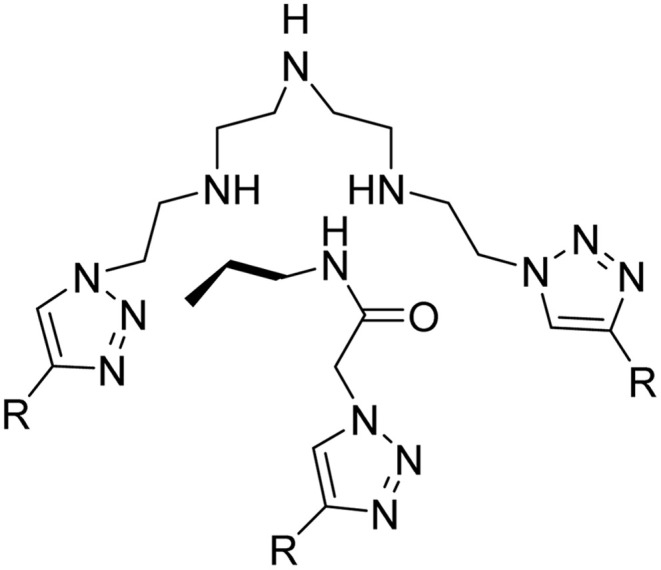	2.87	32
4	BODIPY-based fluorescent probe	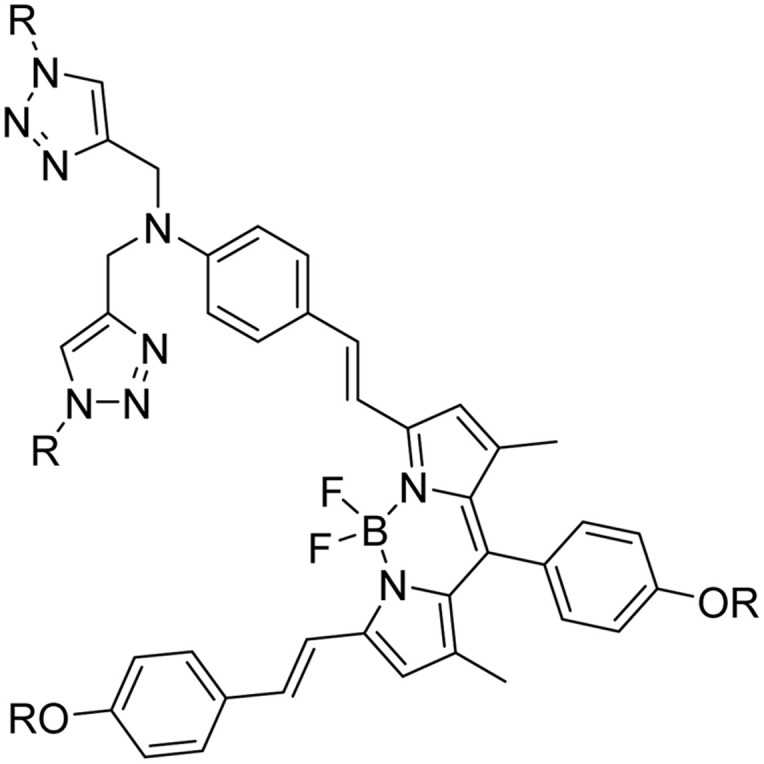	1.02	33
5	Bis-appended 1,2,3-triazole probe	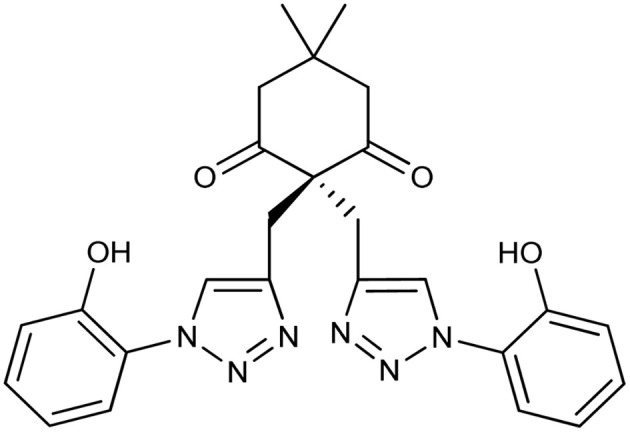	2	6
6	APT	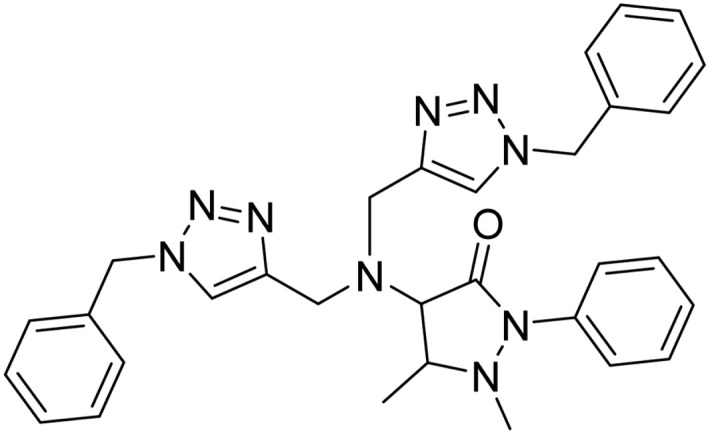	3.11	34
7	Indolin-2-one functionalized siloxy framework	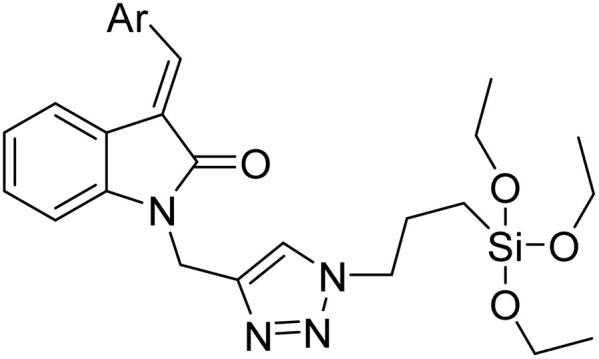	3.21	35
8	Ferrocene–chalcone–triazole	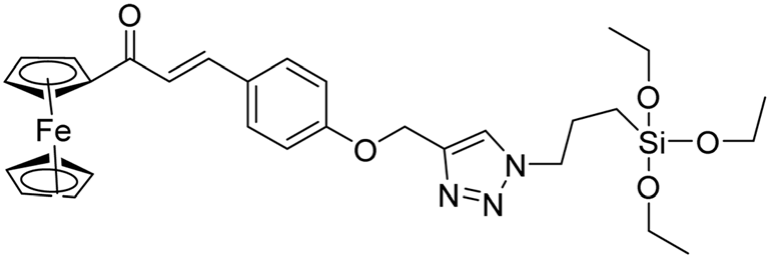	13.04	36
9		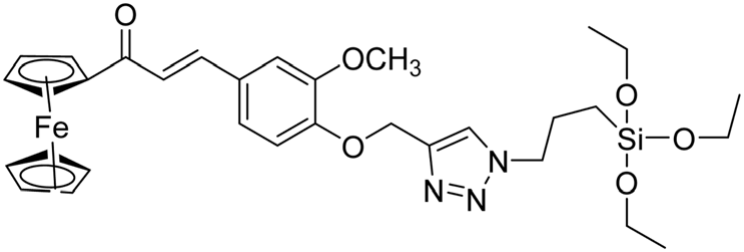	9.42	
10		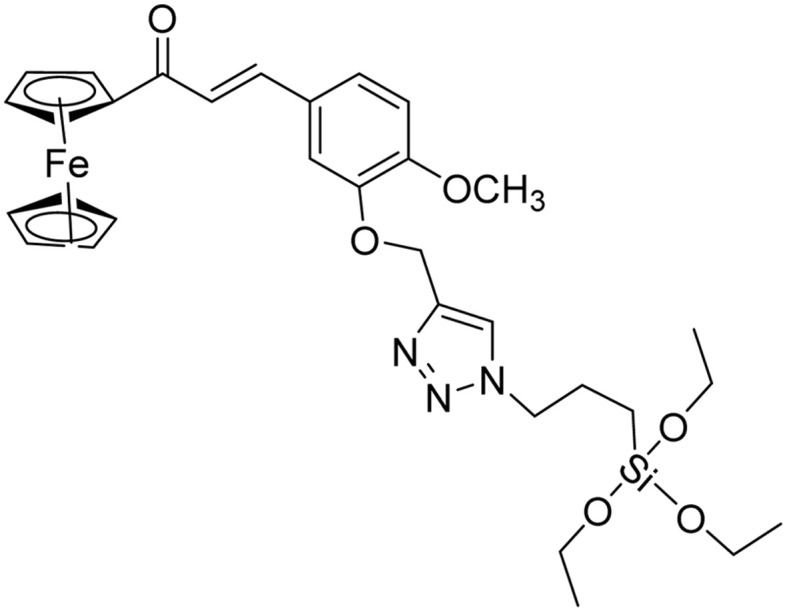	6.70	
11	Probe 6	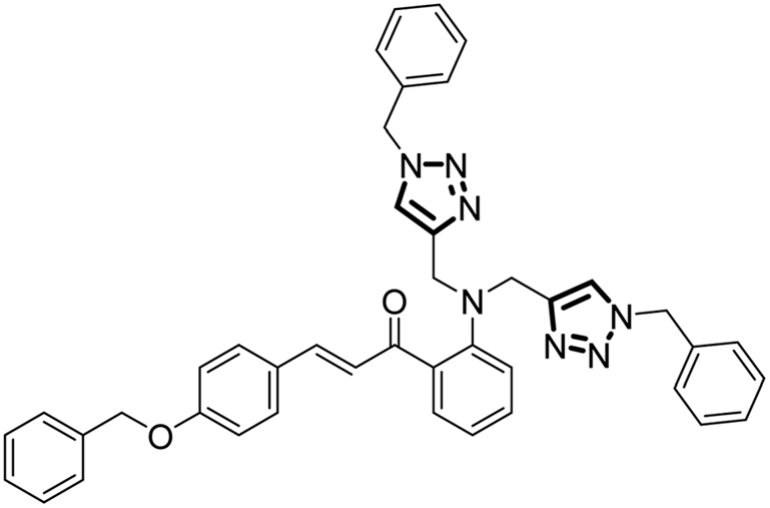	6.55	This work
12	Probe 9	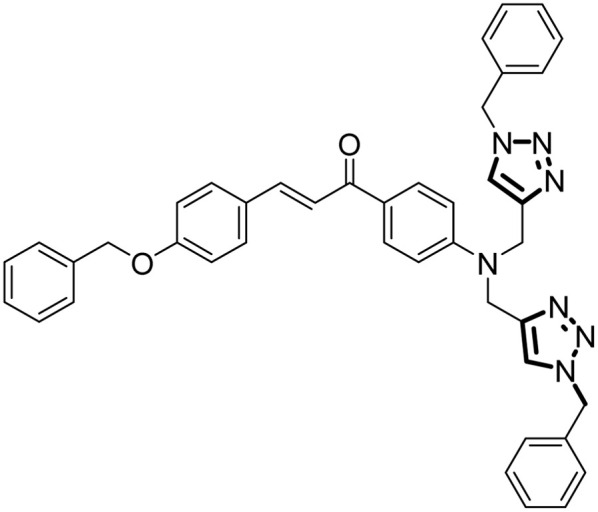	7.52	This work

**Fig. 11:**
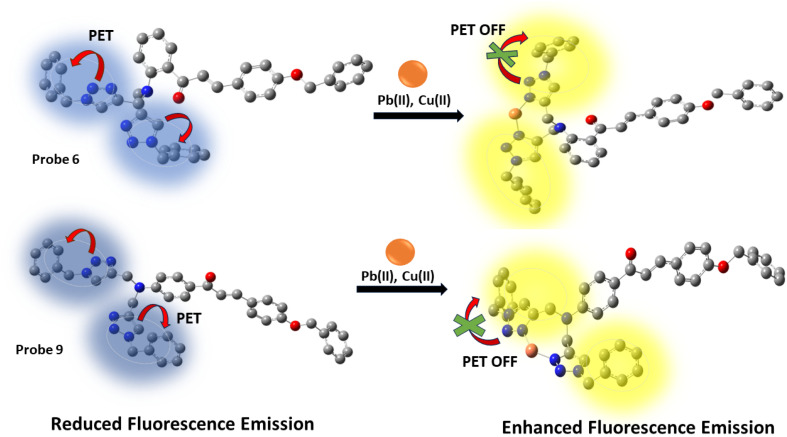
A pictorial representation of photoinduced electron transfer (PET) in probes 6 and 9.

The increased fluorescence emission of probes 6 and 9 when metal ions are added may be explained by the photoinduced electron transfer mechanism. In the absence of metal ions, the nitrogen atoms in the 1,2,3-triazole group, having lone pairs of electrons, may engage in photoinduced electron transfer (PET) even when they are close to benzyl groups. This leads to limited emission, where the non-radiative pathway is the main route for the de-excitation process. However, when metal binds with the nitrogen from the 1,2,3-triazole moiety, the benzyl groups relocate themselves away from the 1,2,3-triazole structure (Fig. 11). This repositioning inhibits the process of PET (photoinduced electron transfer), leading to a considerable increase in fluorescence emission.

### 3.5. Synthesis and confirmation of metal–ligand complex: ^1^H NMR

Following the binding stoichiometry from Job's plot analysis between probes 6 and 9 with the respective metal ions using Job's plot, the metal–ligand complexes were synthesized separately by dissolving probes 6 and 9 and corresponding metal chlorides (1 : 1 molar ratio) in a chloroform/methanol mixture (1 : 1) and subsequently the mixture was allowed to stir for 4 hours. The successful formation of the desired products was indicated by a noticeable change in the color of the reaction solution post-reflux. After filtration, solvent evaporation under vacuum yielded crude products, which were then characterized *via*^1^H NMR analysis. The spectroscopic data, revealed the metal–ligand complexes formation through interaction between the metal atom and the nitrogen atoms of the triazole moieties. This interaction was evidenced by the observed downfield shifts of the peaks corresponding to the 1,2,3-triazole ring protons in the ^1^H NMR spectra of probes 6 and 9 upon complexation with the metal ions. Specifically, for probe 6, the peaks shifted from *δ* = 7.65 ppm to *δ* = 7.77 ppm, while for probe 9, the shifts occurred from *δ* = 7.61 ppm to *δ* = 7.72 ppm. Furthermore, DFT analysis of the metal–ligand complexes was conducted using the 631G+(d,p) basis set for the ligands and the LANL2DZ basis set for the metal atoms. This theoretical analysis corroborated the experimental findings, confirming the presence of binding interactions between the metal atom and the N atoms of the triazole moieties.

## 4. Computational analysis

### 4.1. Structure optimization *via* density functional theory (DFT)

**Fig. 12:**
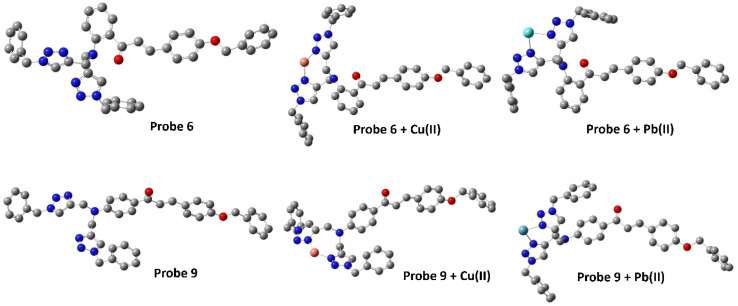
Energy optimized structure of probe 6 and probe 9 using B3LYP/631 G+(d,p) basis set (H atoms have been removed for better clarity).

Density Functional Theory (DFT) serves as a foundational approach in computational chemistry, offering an insightful method for predicting and refining the optimized structures of molecules, materials, and complex systems by the employment of quantum mechanical equations governing the electron density distribution.^37^ Envisaging this, the energy-optimized structures of both probes 6 and 9 were investigated using the B3LYP hybrid functional with the basis set 631G+(d,p) in DFT (Gaussian software).^38^ The optimized structures of the probes 6 and 9 are illustrated in Fig. 12 (cartesian coordinates provided in ESI, Table S1, and S2†). Furthermore, the binding interactions of both the probes with the sensed metal ions were also investigated using the LANL2DZ basis set for the metal ions. The probes can bind with the metal ions *via* the lone-pair bearing N atoms of both the 1,2,3-triazole moieties. This theoretical binding mode is further supported by the ^1^H NMR and mass of the metal–ligand complex.

### 4.2. Frontier molecular orbitals investigation

**Fig. 13:**
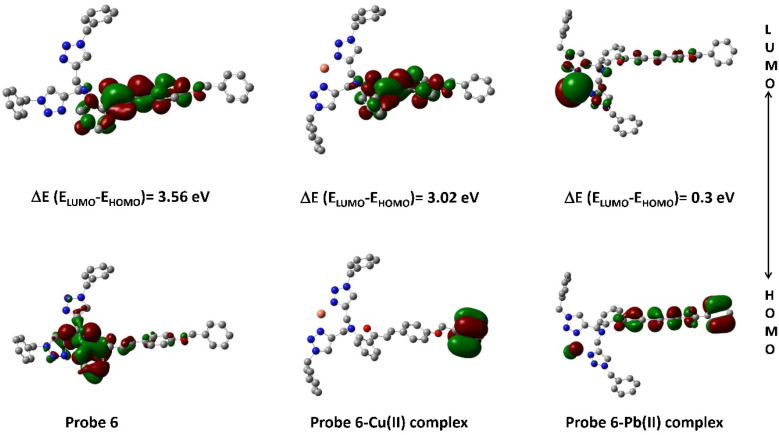
Density plot depicting the HOMO–LUMO interaction between probe 6 and its respective metal ions.

Within the realm of DFT, the Highest Occupied Molecular Orbital (HOMO) and Lowest Unoccupied Molecular Orbital (LUMO) emerge as pivotal entities, playing a profound role in unraveling the electronic structure and reactivity of molecules and materials. Fig. 13 depicts the density plots of the HOMO and the LUMO of probe 6 and its corresponding metal complex. As can be observed, the HOMO of probe 6 is mostly delocalized on the 1,2,3-triazole moieties and its adjoining benzyl of the parent chalcone body, while the LUMO is delocalized on the aromatic rings of the parent chalcone structure only. In the case of the Cu(ii) complex of probe 6, it is observed that the HOMO remains condensed over the ‘tail-end’ aromatic ring of the chalcone body whereas the LUMO gets delocalized over the other two aromatic rings of the chalcone moiety. The Pb(ii) complex demonstrates different delocalization, with the HOMO being delocalized over the chalcone structure and the LUMO being delocalized mostly over the Pb(ii) ion and the 1,2,3-triazole moieties.

**Fig. 14:**
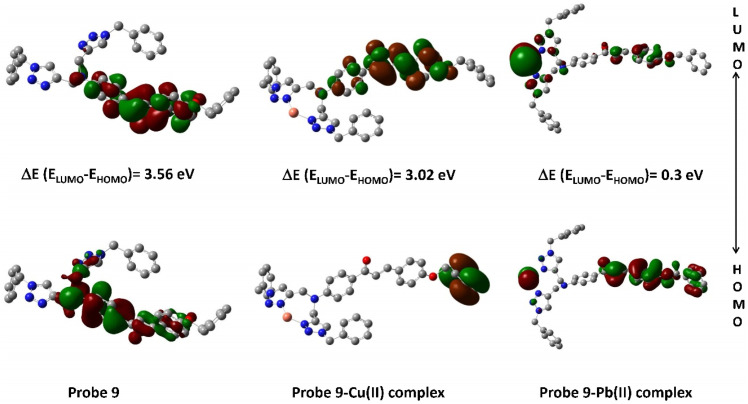
Density plot depicting the HOMO–LUMO interaction between probe 9 and its respective metal ions.

The density plots of probe 9 and its corresponding complex are illustrated in Fig. 14, wherein it is exhibited that the HOMO is mostly delocalized over one of the 1,2,3-triazole moieties and the chalcone body excluding the ‘tail-end’ aromatic ring, while the LUMO is confined to the chalcone structure only, again without the participation of the ‘tail-end’ aromatic ring. In the case of the Pb(ii) complex, the HOMO is mostly delocalized over the chalcone structure whereas the LUMO is delocalized over the Pb(ii) ion in addition to the 1,2,3-triazole moieties.

### 4.3. Molecular electrostatic potential (MEP)

**Fig. 15:**
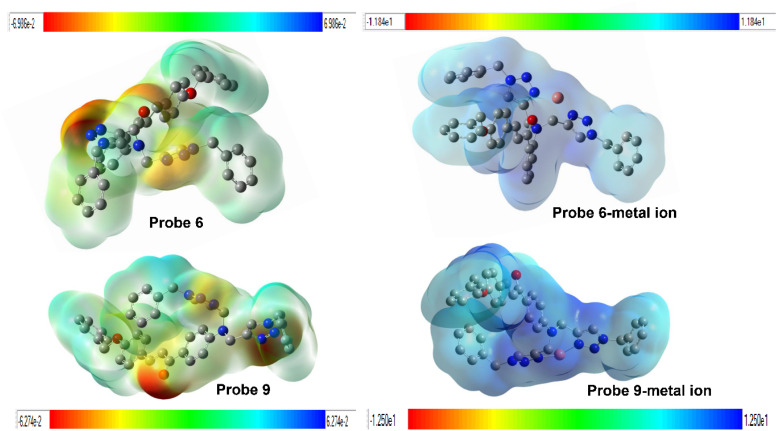
A pictorial representation of molecular electrostatic potential (MEP) map for probes (6, 9) and their interaction with the metal ions.

To anticipate the molecule's reactivity, it is essential to identify its electrophilic and nucleophilic regions.^39^ The molecular electrostatic potential is used to determine the specific places in the molecule where attacks occur, depending on the distinction of colors.^40^ The color bar represents the range of possible values from the most negative to the most positive on the MEP scale, with red indicating the most negative values and blue indicating the most positive ones. The red hue has the maximum electrostatic value, which gradually decreases towards the blue color. The presence of electron-rich triazole rings and a carbonyl group in Probes 6 and 9 were seen as red, suggesting their capacity for intermolecular interactions and their negative electrostatic potential, as shown in Fig. 15. The interaction between probes 6 and 9 and metal ions results in a decrease in electron density at the nitrogen atoms of the triazole ring.

## 5. Plausible binding mode

**Fig. 16:**
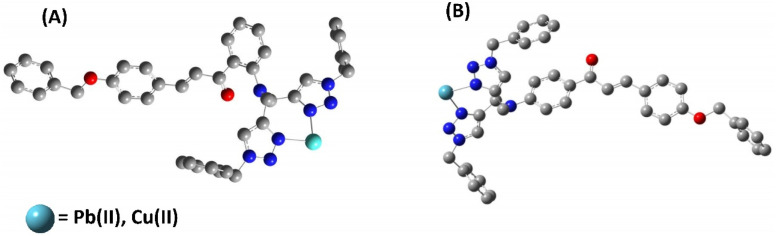
Potential binding of the (A) probe 6 with the metal ions (B) probe 9 with metal ions (H atoms have been removed for clarity).

According to the hard soft acid base (HSAB) principle, Pb(ii) and Cu(ii) are considered to exhibit characteristics of weak acids.^41^ Metal ions can potentially interact with atomic groups that possess lone pairs, such as N, O, or S.^42^ Based on the HSAB theory, the probes can efficiently seize electron-deficient metal ions through interaction with the nitrogen atoms carrying lone pairs in the 1,2,3-triazole groups. Furthermore, the ion detection property of the sensor relies on a host–guest interaction, where the receptor is linked to the fluorophore by providing a suitable-sized cavity for the incoming metal ion and facilitates the recognition process. The host component is either covalently or coordinately bonded with the guest module, and upon complex formation with the target metal ion, it induces change in the emission intensity.^17^ The job's plot for the probe demonstrates the formation of a 1 : 1 complex between the metal and ligand. Furthermore, the N atoms of the 1,2,3-triazole moiety were found to be involved in binding the metal ions, as confirmed by ^1^H NMR spectra of the probe 6-metal complex and probe 9-metal complex, along with DFT calculations. The selectivity of both probes for Pb(ii) over the other metal ions may be ascribed to the receptor cavity's appropriate size for Pb(ii). Based on these data, a hypothesized binding mechanism between the probe and the metal ions is shown in Fig. 16, demonstrating the metal–ligand interaction seen in the results.

## 6. Conclusion

The chalcone stitched bis-1,2,3-triazole chemosensors were synthesized using CuAAC methodology serving as potential ion recognition agents to detect the toxic and environmentally threatening metal ions specifically Cu(ii) and Pb(ii) with high selectivity towards these metal ions through absorption and fluorescence spectroscopy. The probes exhibited time and temperature-invariant characteristics, as shown by their ability to bind to Pb(ii) and Cu(ii) ions. The strong and stable binding of both probes is evidenced by a linear relationship with no variation in binding affinity over time. The association constant values obtained from the Benesi–Hildebrand (B–H) plot demonstrated the superior binding affinity of both probes with Pb(ii) ions. This finding substantiates the outcomes of competitive metal ion titration experiments, affirming the probes' ability to selectively detect Pb(ii) ions even in the presence of various other metal ions. Furthermore, the distinctive molecular configuration of the probes 6 and 9 as well as their interaction with the respective aforementioned metal ions was determined by DFT calculation.

## Author contributions

Riddima Singh: writing – original draft, investigation, formal analysis. Gurleen Singh: data curation, methodology, resources. Nancy George: data curation, methodology. Gurjaspreet Singh: visualization, validation. Pooja Malik: formal analysis, resources. Harminder Singh: conceptualization, validation. Gurpreet Kaur: investigation, writing– review & editing, supervision. Jandeep Singh: conceptualization, supervision.

## Conflicts of interest

There are no conflicts to declare.

## Supplementary Material

RA-014-D4RA01471E-s001
