# Supplementary material for: Unveiling the ion sensing capabailities of ‘click’ derived chalcone-tailored 1,2,3-triazolic isomers for Pb(ii) and Cu(ii) ions: DFT analysis
Source: RSC Adv. 2024 May 13;14(22):15374–90. doi: 10.1039/d4ra01471e (PMC11089528; doi:10.1039/d4ra01471e)
Supplement: RA-014-D4RA01471E-s001 [file RA-014-D4RA01471E-s001.pdf]

Electronic Supplementary Material (ESI) for RSC Advances.
This journal is © The Royal Society of Chemistry 2024

Supporting Information

Riddima Singh,[a] Gurleen Singh,[a] Nancy George,[a] Gurjaspreet Singh,[b] Pooja Malik,[b] Harminder Singh,[a] Gurpreet Kaur,[c,#] Jandeep Singh,[a,*]

[a] School of Chemical Engineering and Physical Sciences, Lovely Professional University, Phagwara–144411 (Punjab)

[b] Department of Chemistry and Centre of Advanced Studies in Chemistry, Panjab University, Chandigarh–160014

[c] Department of Chemistry, GGN Khalsa College, Civil lines, Ludhiana–141001 (Punjab)

***,# Corresponding Authors**

Contact details:

***singhjandeep@gmail.com**

Contents:

Figure S1: IR spectrum of chalcone 4

FigureS2: IR spectrum of chalcone 7

Figure S3: IR spectrum of alkyne 5

Figure S4: IR spectrum of alkyne 8

Figure S5: IR spectrum of benzyl azide 10

Figure S6: IR spectrum of probe 6

Figure S7: IR spectrum of probe 9

Figure S8: ^{1}H NMR spectrum of alkyne 5

Figure S9: ^{13}C NMR spectrum of alkyne 5

Figure S10: ^{1}H NMR spectrum of alkyne 8

Figure S11: ^{13}C NMR spectrum of alkyne 8

Figure S12: ^{1}H NMR spectrum of benzyl azide 10

Figure S13: ^{13}C NMR spectrum of benzyl azide 10

Figure S14: ^{1}H NMR spectrum of probe 6

Figure S15: ^{13}C NMR spectrum of probe 6

Figure S16: ^{1}H NMR spectrum of probe 9

Figure S17: ^{13}C NMR spectrum of probe 9

Figure S18: Mass spectrum of probe 6

Figure S19: Mass spectrum of probe 9

Figure S20: Time dependent spectra of probe 6-Pb(II) complex displaying the trend in the absorption.

Figure S21: Time dependent spectra of probe 6-Cu(II) complex displaying the trend in the absorption.

Figure S22: Time dependent spectra of probe 9-Pb(II) complex displaying the trend in the absorption.

Figure S23: Time dependent spectra of probe 9-Cu(II) complex displaying the trend in the absorption.

Figure S24: Temperature dependent spectra of probe 6-Pb(II) complex displaying the trend in the absorption.

Figure S25: Temperature dependent spectra of probe 6-Cu(II) complex displaying the trend in the absorption.

Figure S26: Temperature dependent spectra of probe 9-Pb(II) complex displaying the trend in the absorption.

Figure S27: Temperature dependent spectra of probe 9-Cu(II) complex displaying the trend in the absorption.

Figure S28: Job's plot analysis of probe 6 on interaction with Pb(II) ions.

Figure S29: Job's plot analysis of probe 6 on interaction with Cu(II) ions.

Figure S30: Job's plot analysis of probe 9 on interaction with Pb(II) ions.

Figure S31: Job's plot analysis of probe 9 on interaction with Cu(II) ions.

Figure S32: ^{1}H-NMR of probe 6 on interaction with the metal ion.

Figure S33: ^{1}H-NMR of probe 9 on interaction with the metal ion.

Table S1: Cartesian co-ordinates of probe 6

Table S2: Cartesian co-ordinates of probe 9

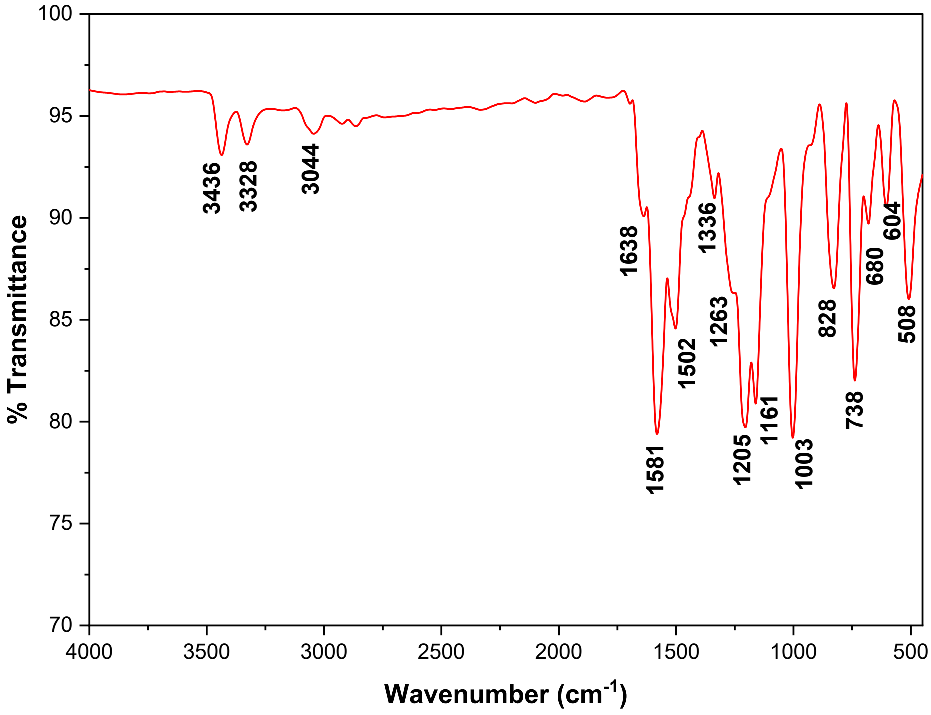

Figure S1: IR spectrum of chalcone 4

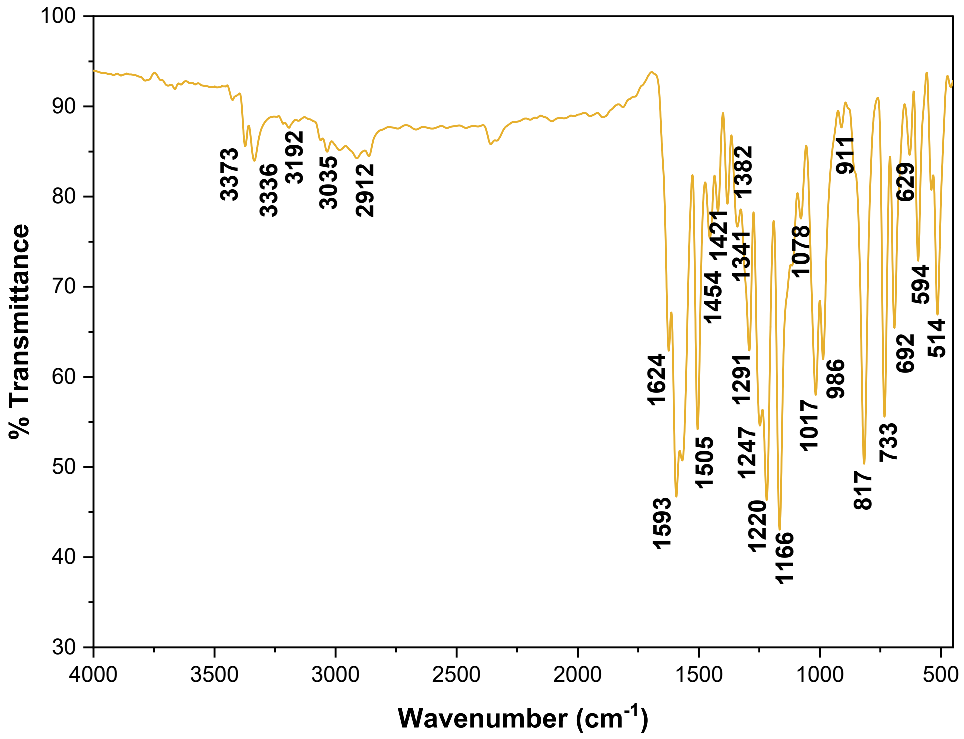

FigureS2: IR spectrum of chalcone 7

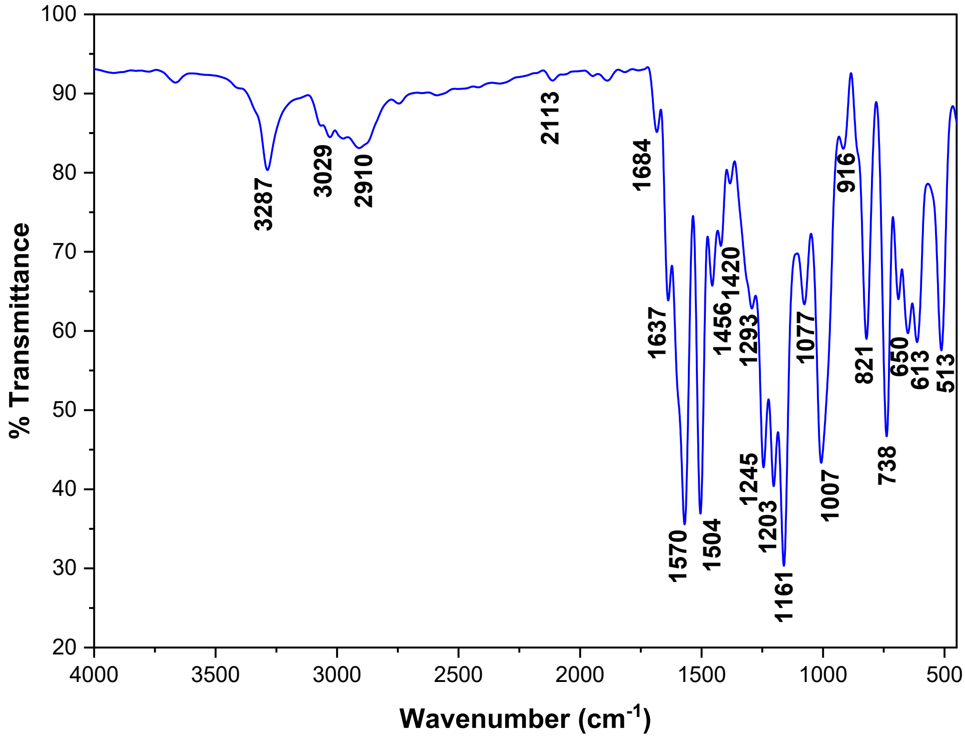

Figure S3: IR spectrum of alkyne 5

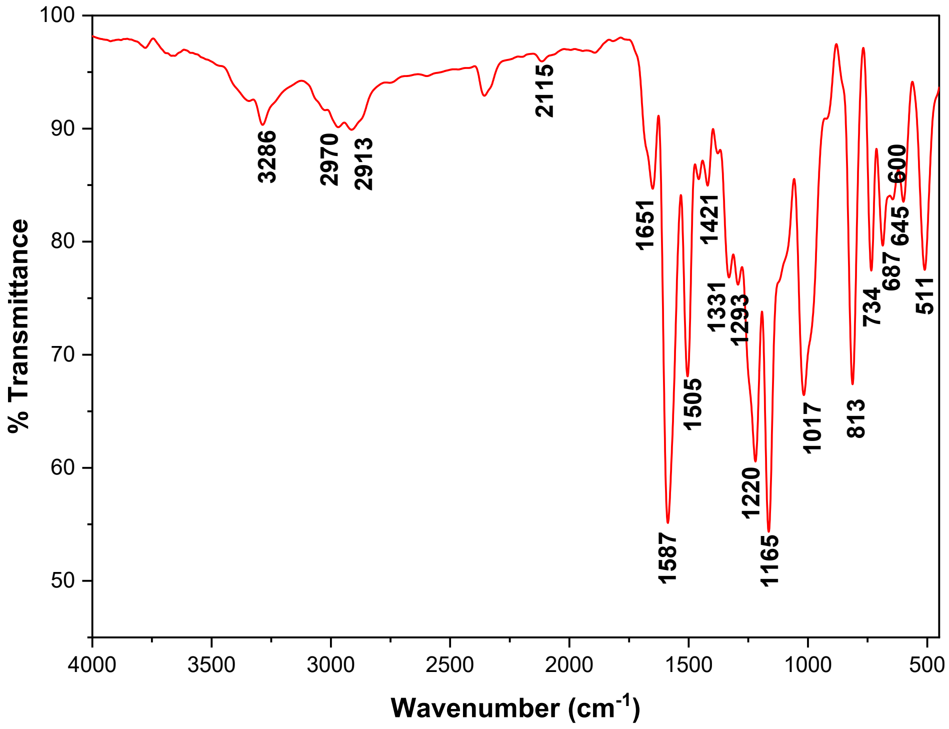

Figure S4: IR spectrum of alkyne 8

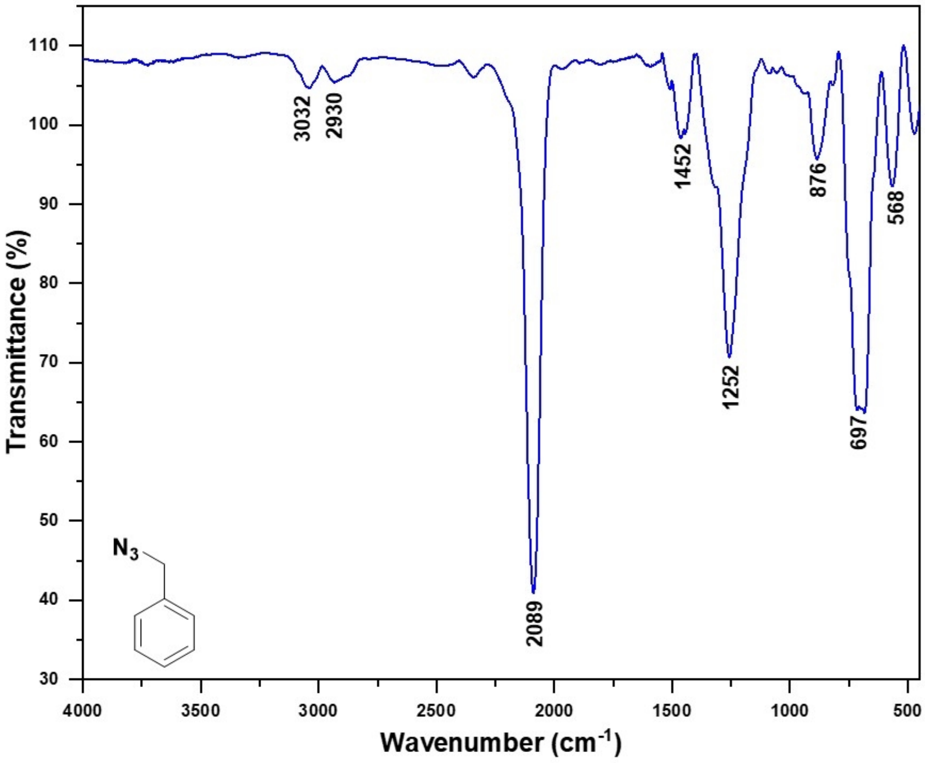

Figure S5: IR spectrum of benzyl azide 10

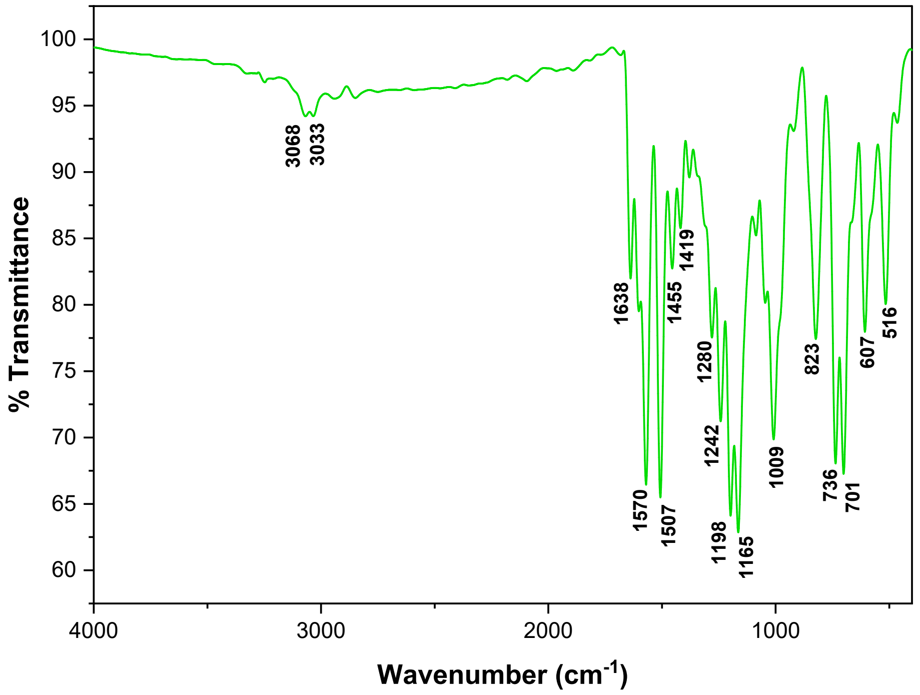

Figure S6: IR spectrum of probe 6

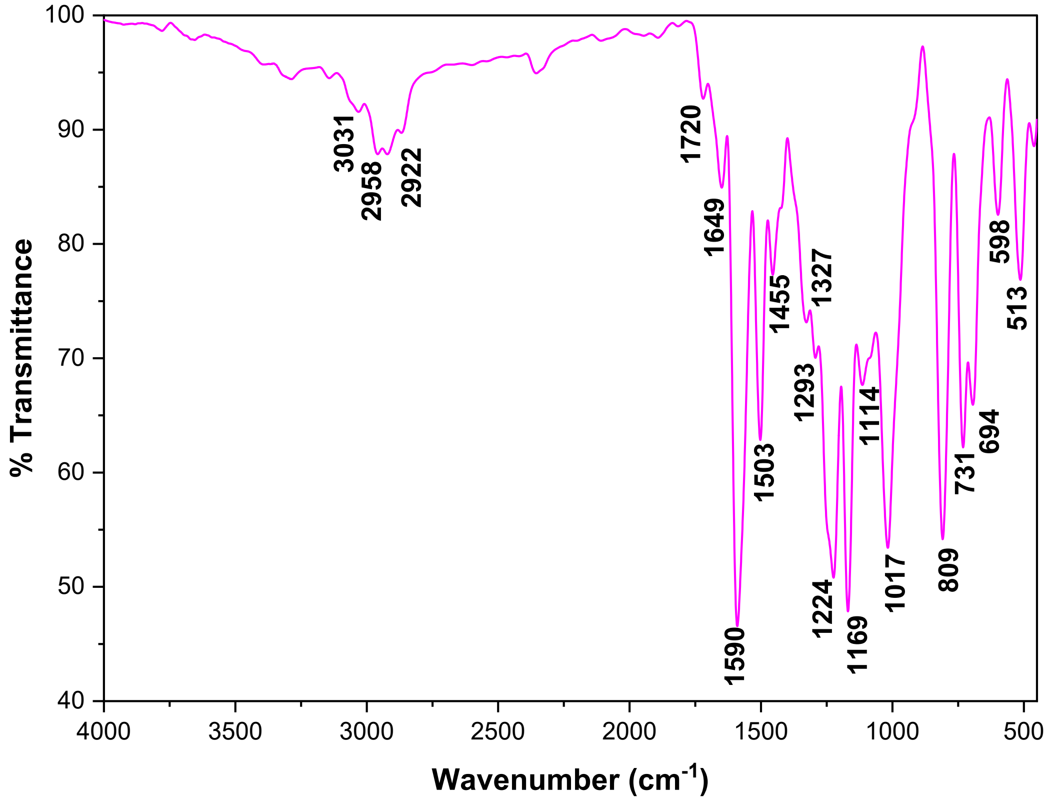

Figure S7: IR spectrum of probe 9

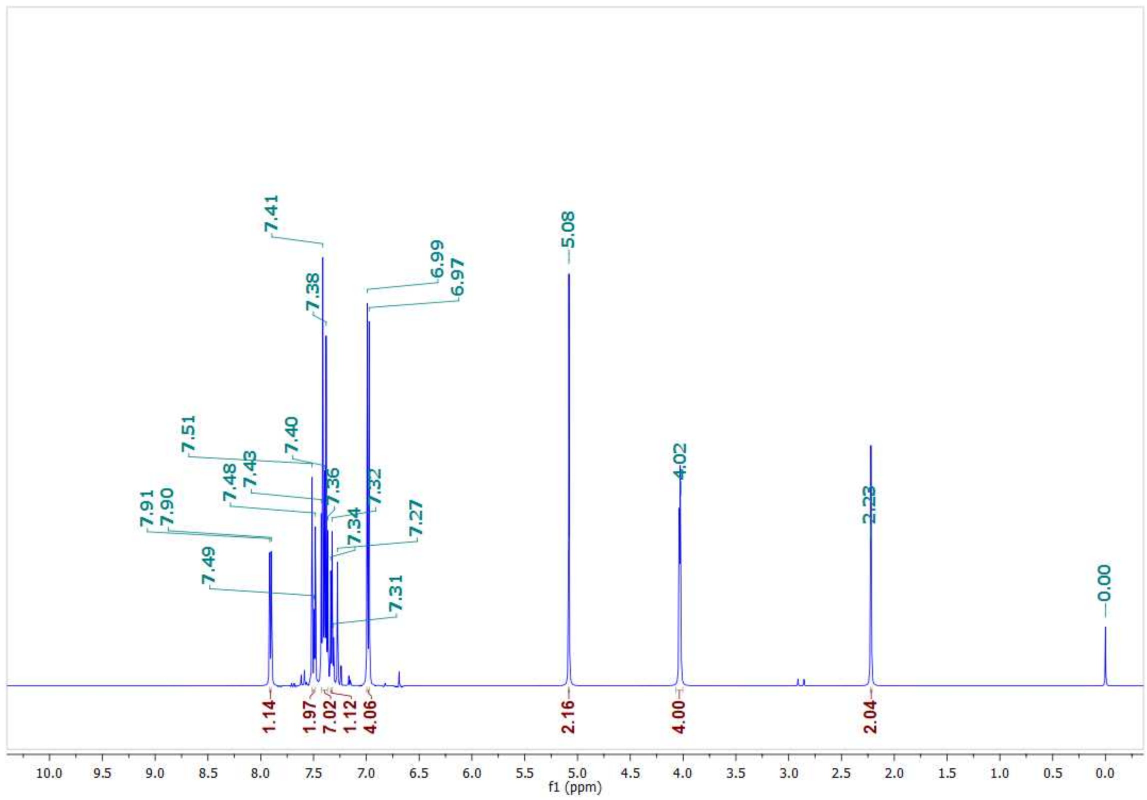

Figure S8: ^{1}H NMR spectrum of alkyne 5

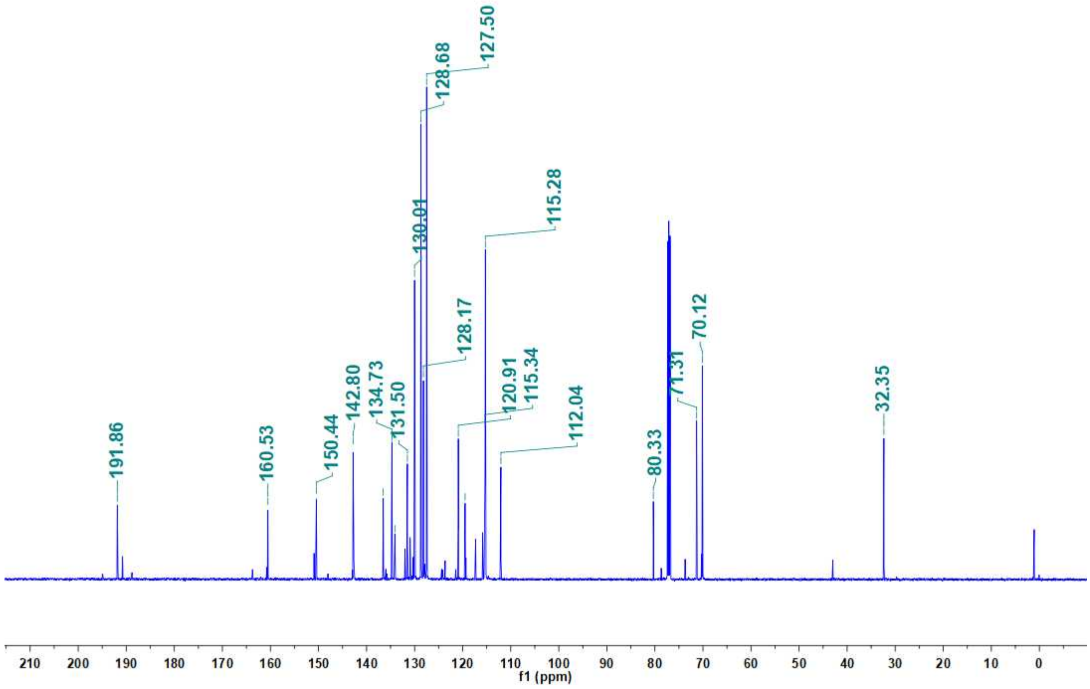

Figure S9: ^{13}C NMR spectrum of alkyne 5

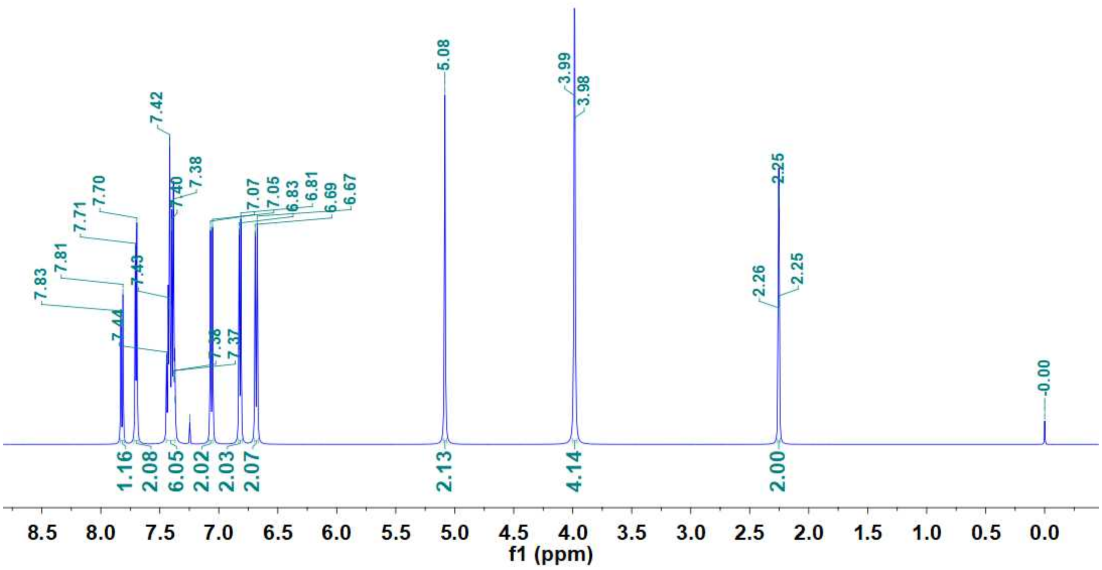

Figure S10: ^{1}H NMR spectrum of alkyne 8

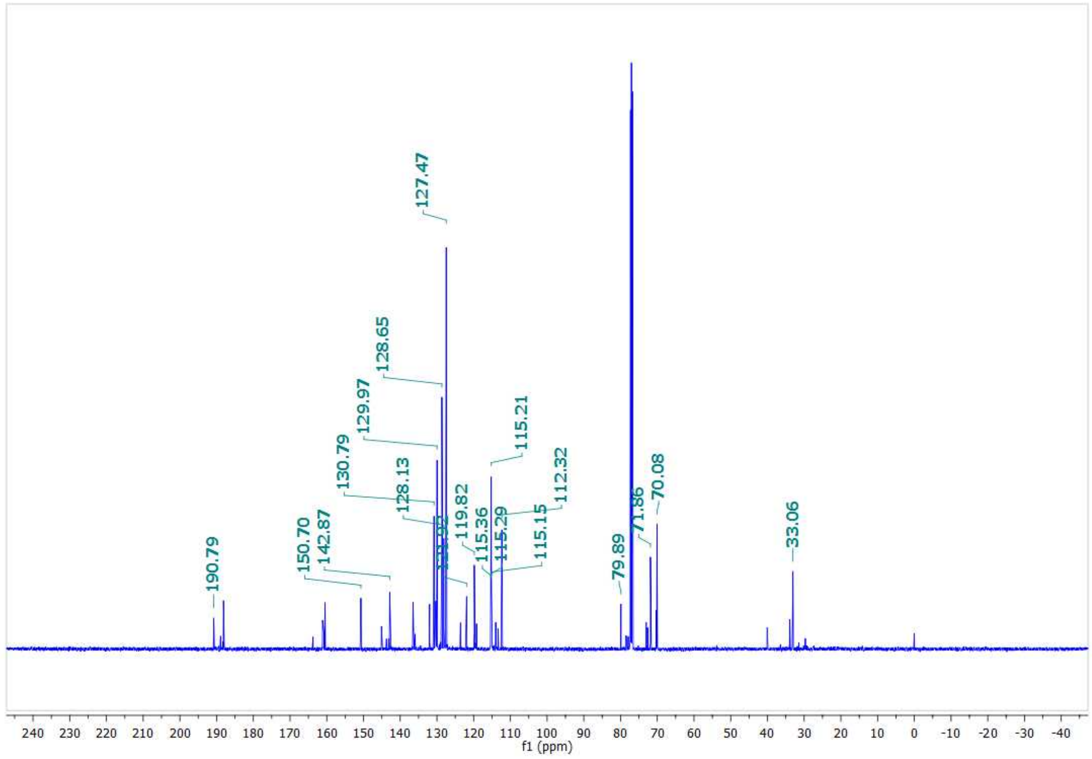

Figure S11: ^{13}C NMR spectrum of alkyne 8

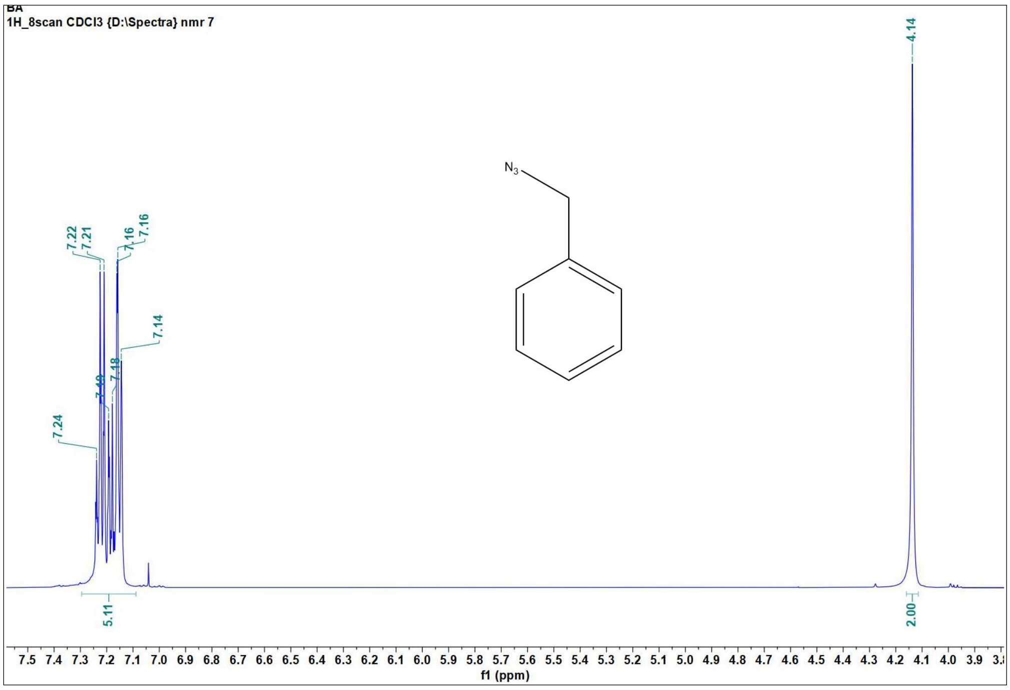

Figure S12: ^{1}H NMR spectrum of benzyl azide 10

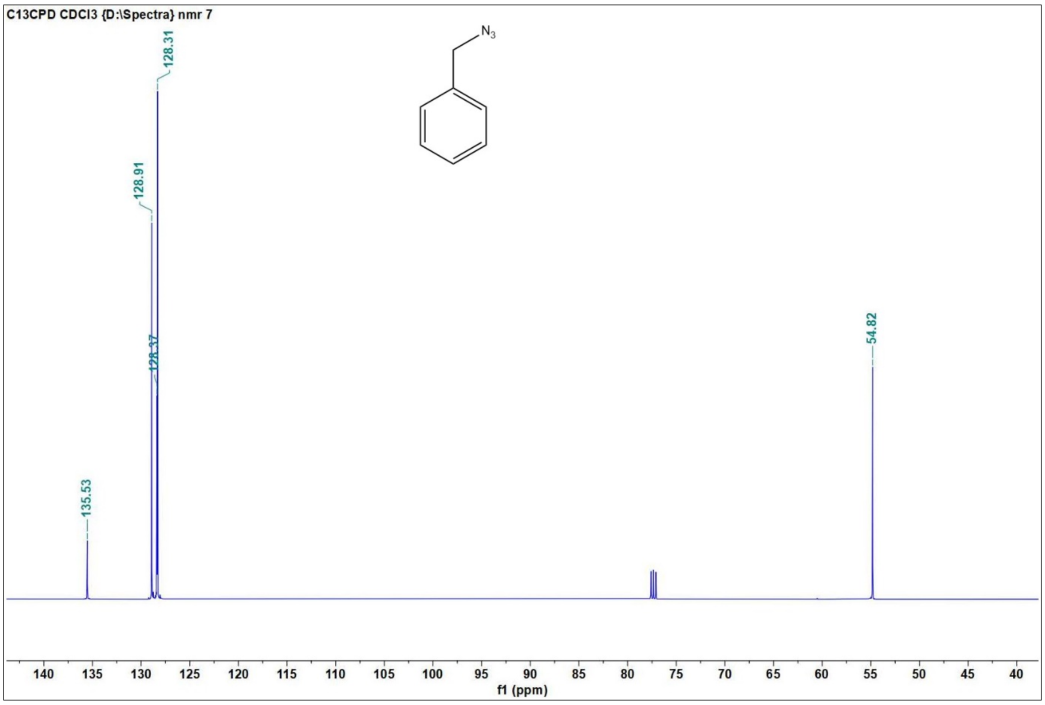

Figure S13: ^{13}C NMR spectrum of benzyl azide 10

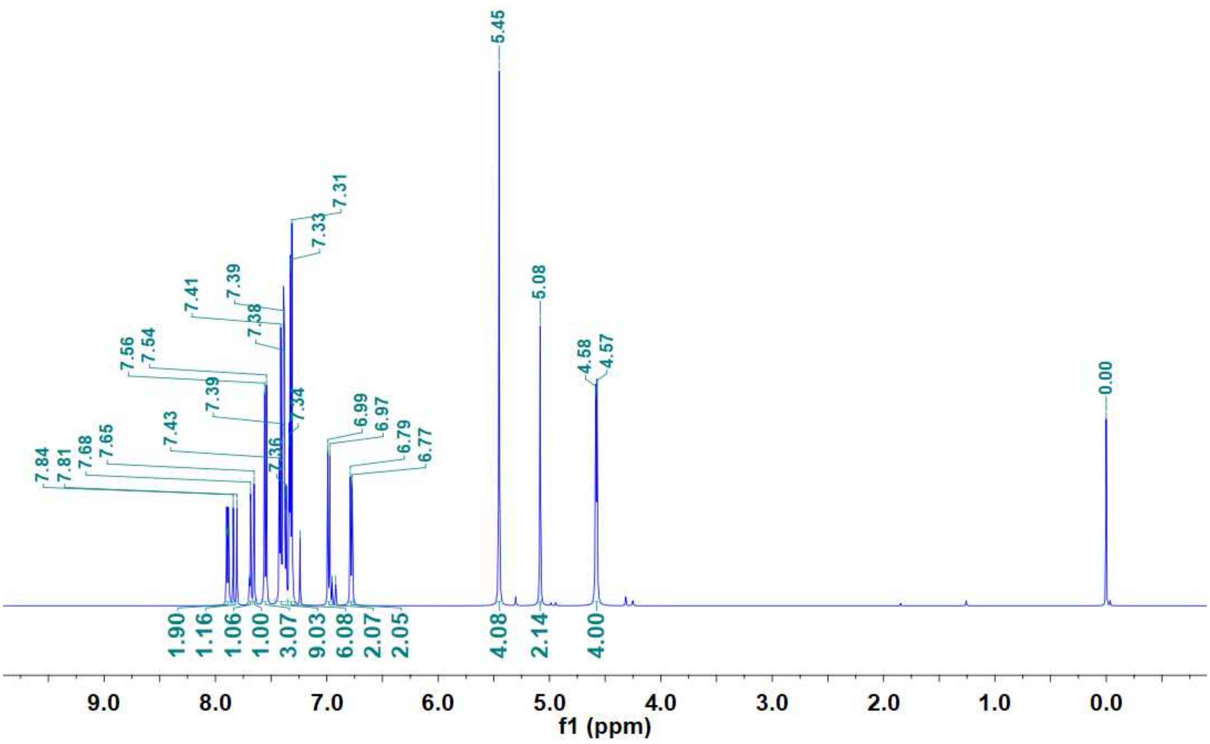

Figure S14: ^{1}H NMR spectrum of probe 6

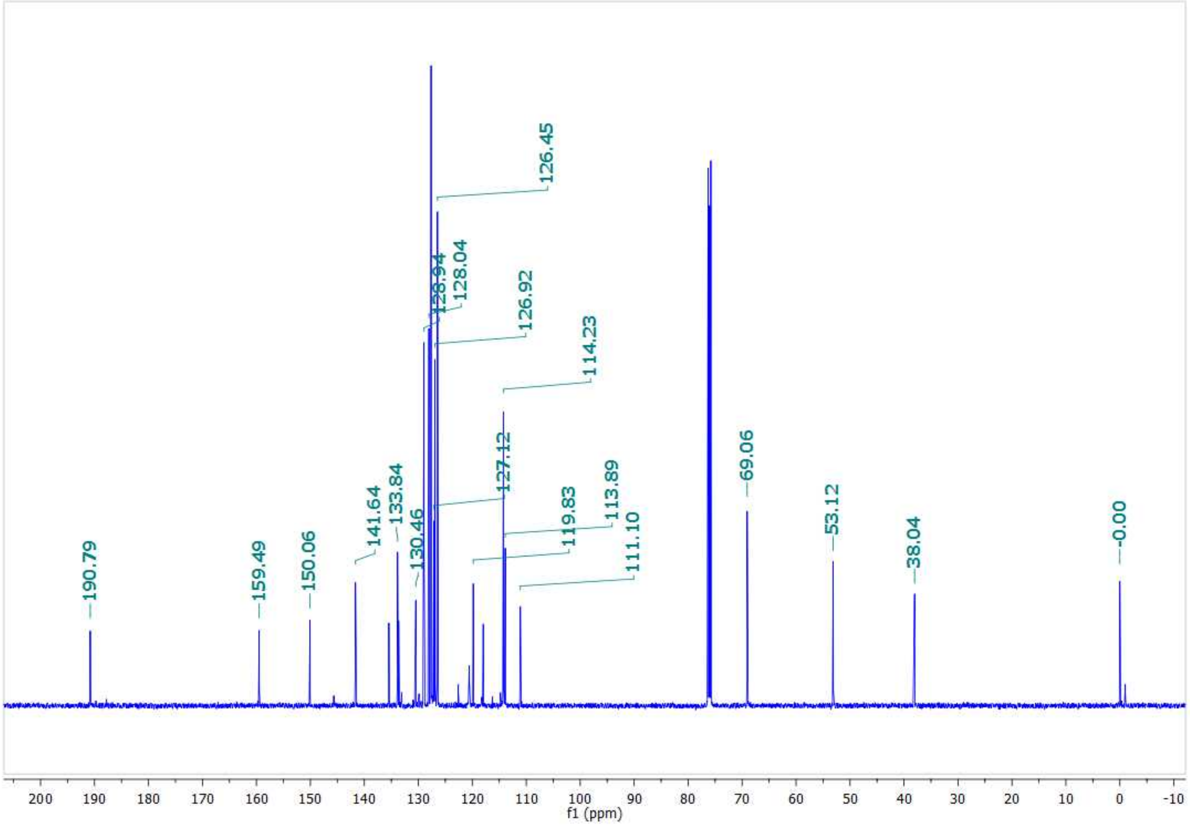

Figure S15: ^{13}C NMR spectrum of probe 6

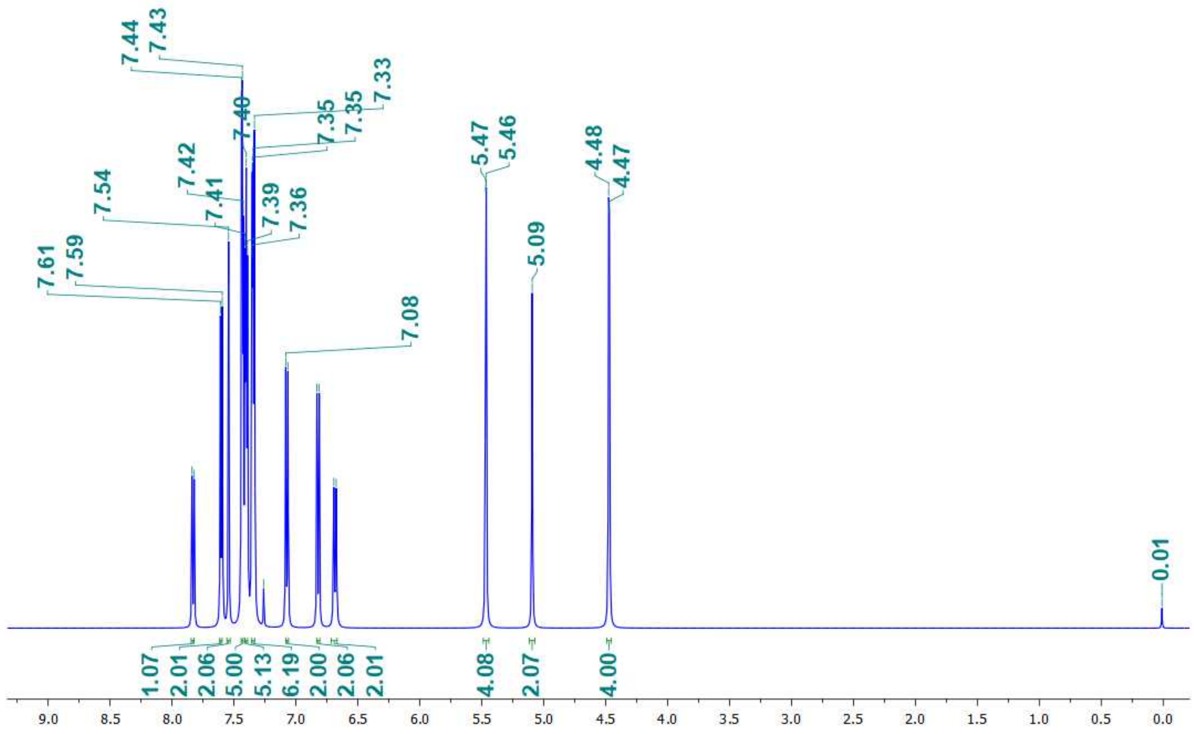

Figure S16: ^{1}H NMR spectrum of probe 9

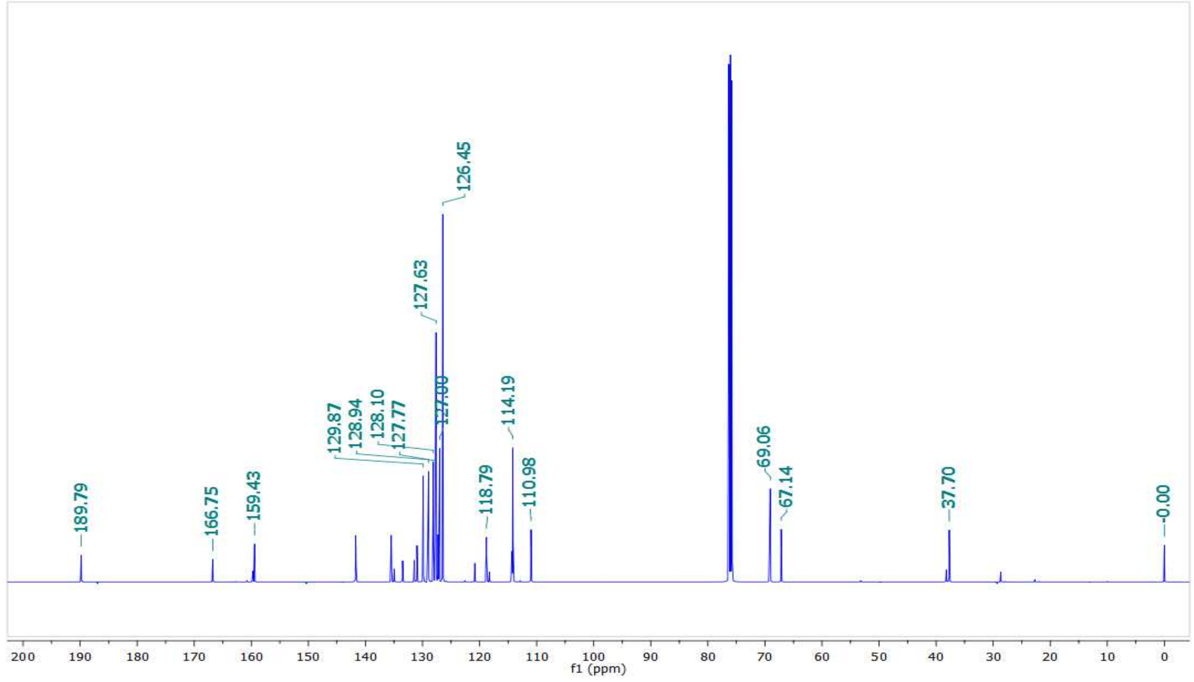

Figure S17: ^{13}C NMR spectrum of probe 9

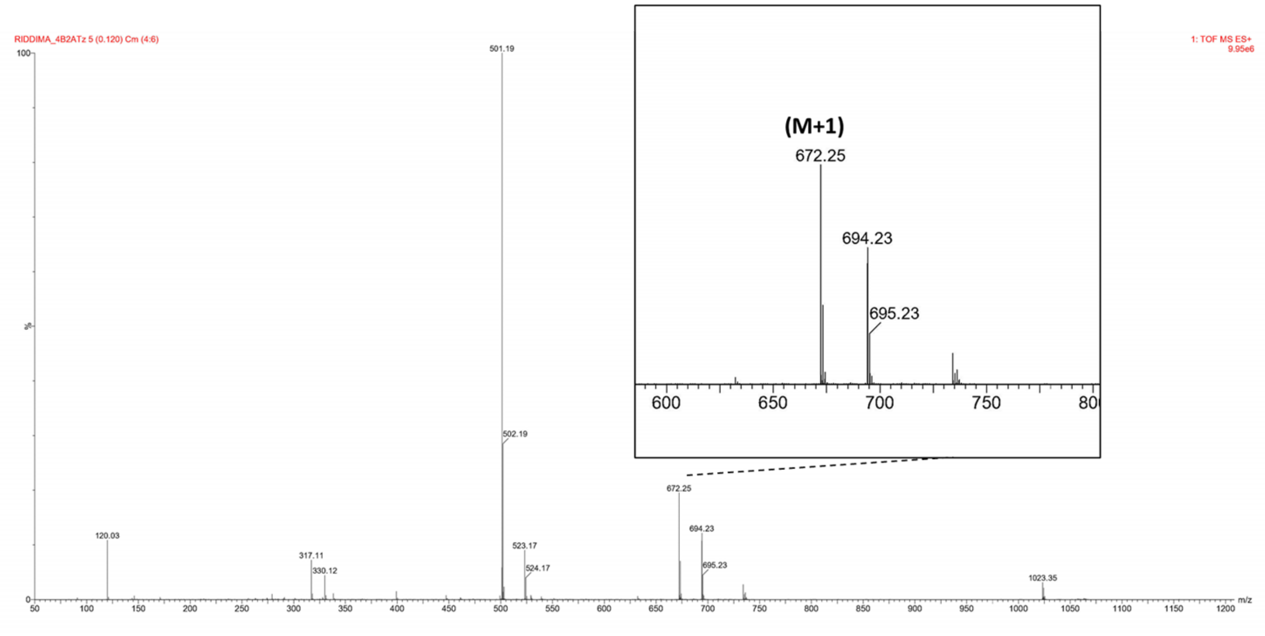

Figure S18: Mass spectrum of probe 6

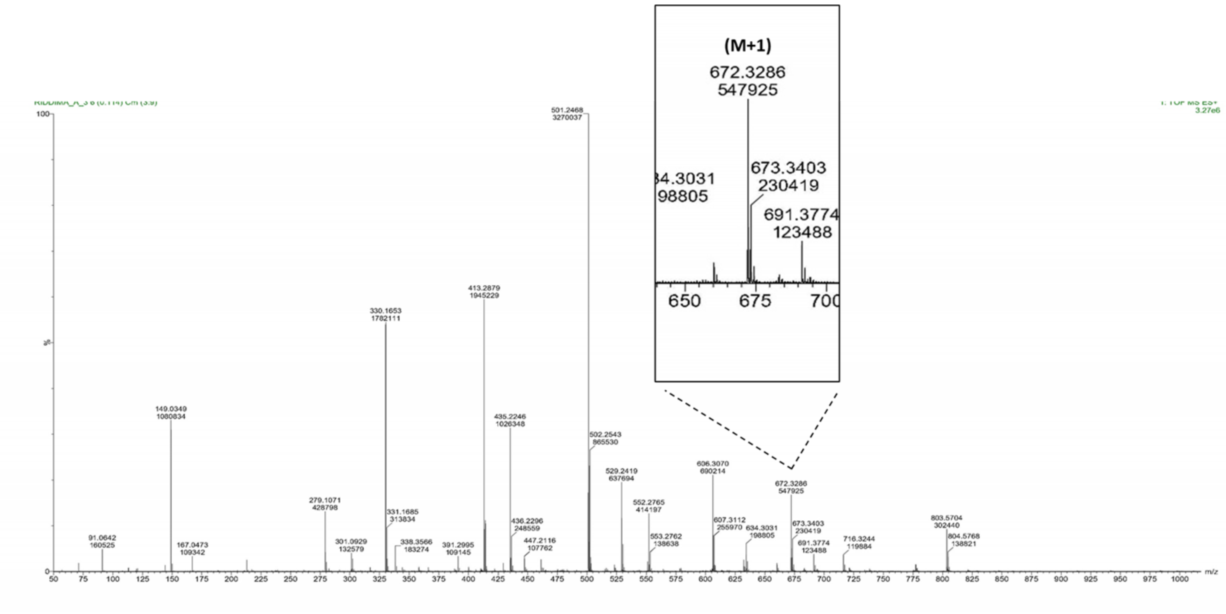

Figure S19: Mass spectrum of probe 9

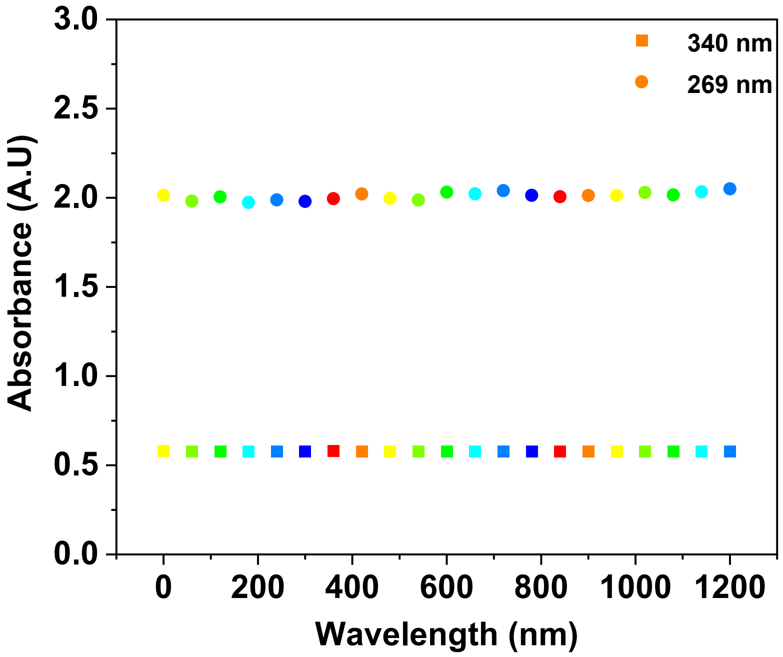

Figure S20: Time dependent spectra of probe 6-Pb(II) complex displaying the trend in the absorption.

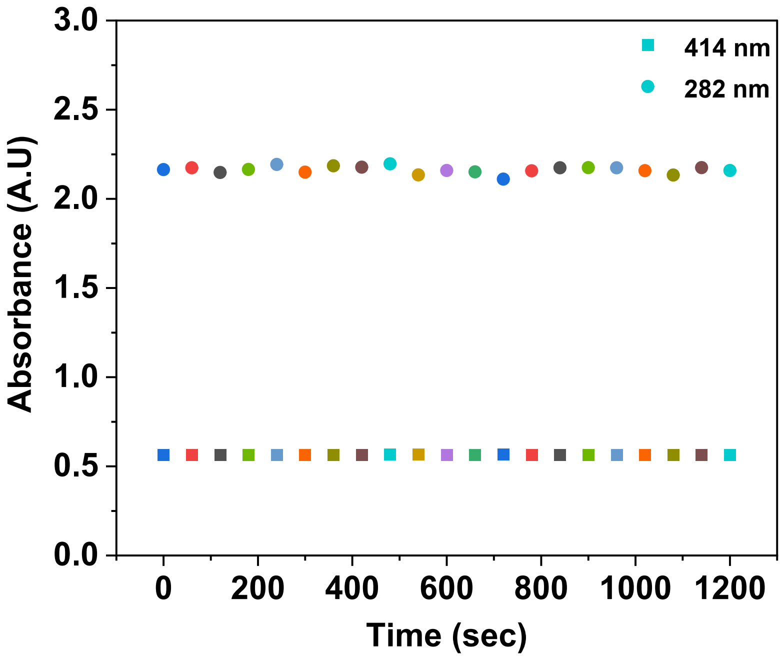

Figure S21: Time dependent spectra of probe 6-Cu(II) complex displaying the trend in the absorption.

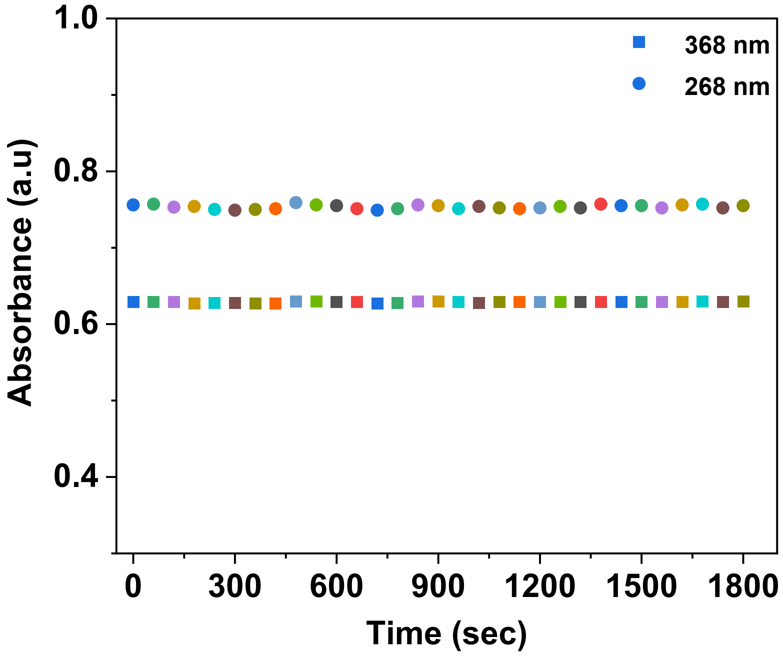

Figure S22: Time dependent spectra of probe 9-Pb(II) complex displaying the trend in the absorption.

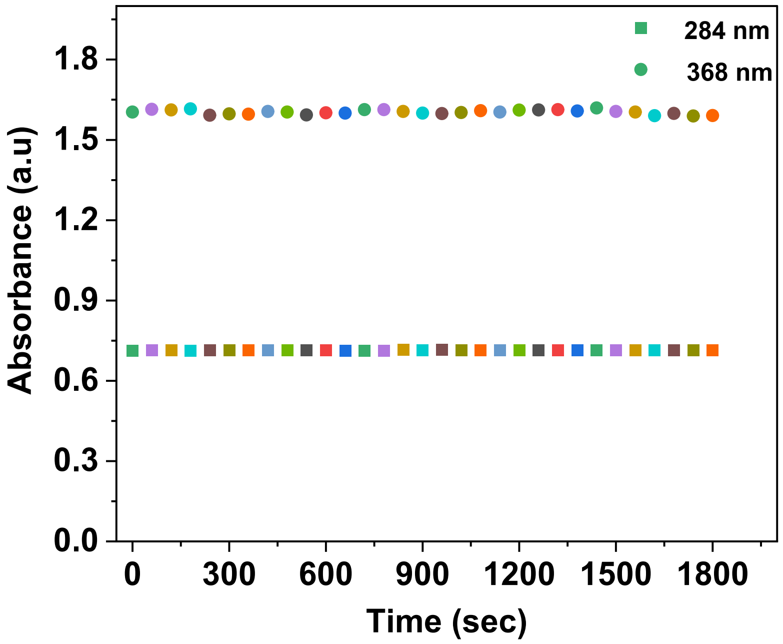

Figure S23: Time dependent spectra of probe 9-Cu(II) complex displaying the trend in the absorption.

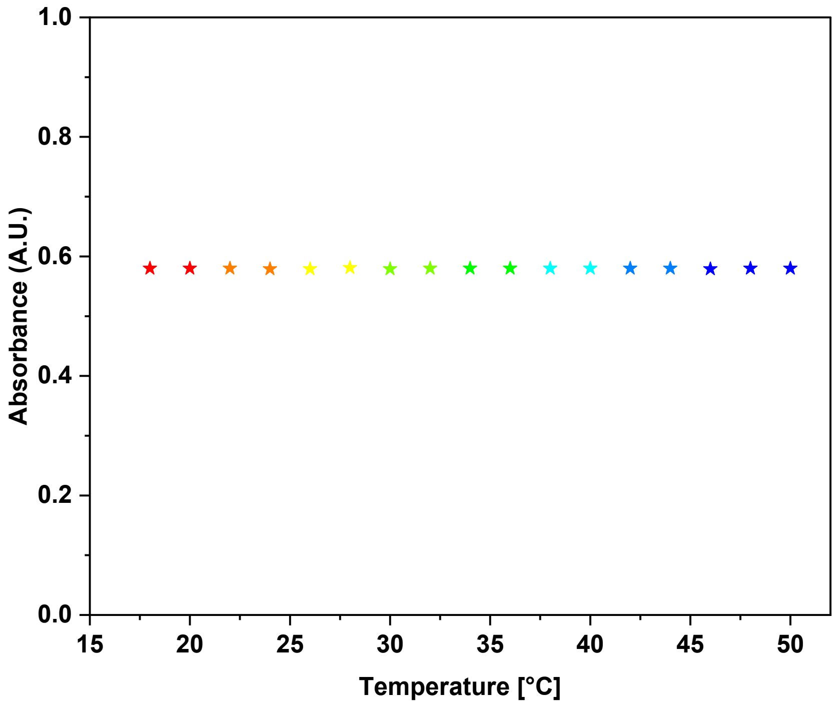

Figure S24: Temperature dependent spectra of probe 6-Pb(II) complex displaying the trend in the absorption.

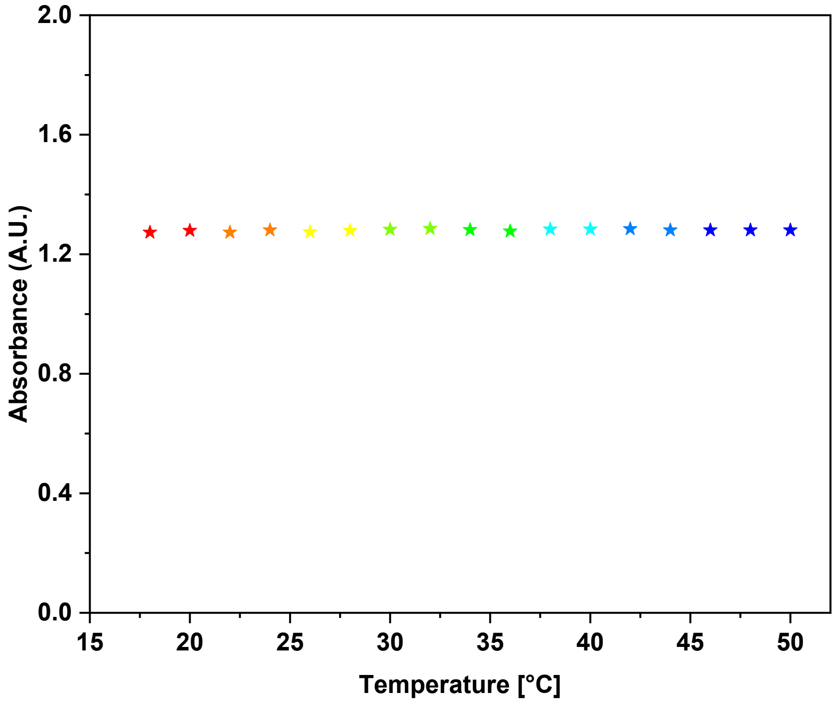

Figure S25: Temperature dependent spectra of probe 6-Cu(II) complex displaying the trend in the absorption.

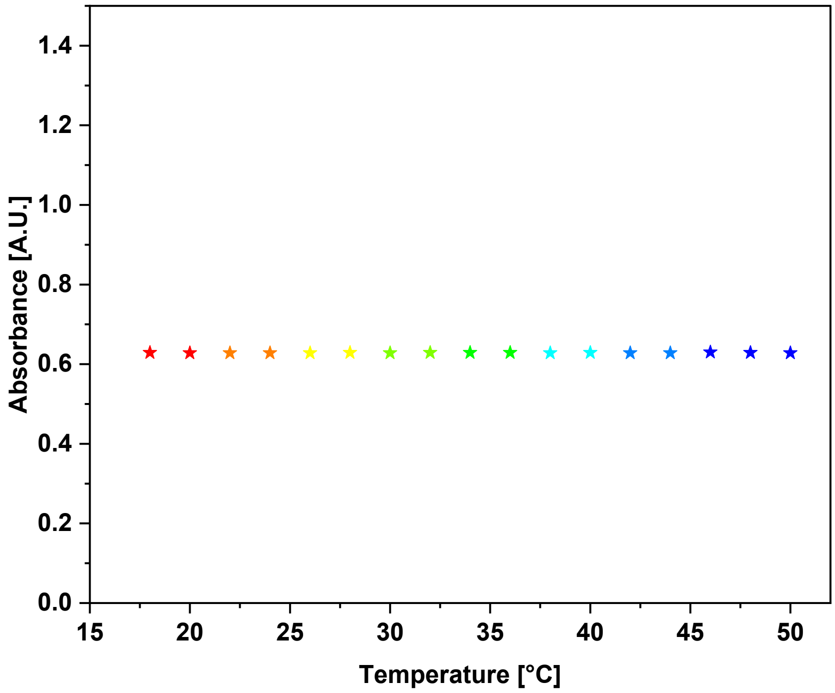

Figure S26: Temperature dependent spectra of probe 9-Pb(II) complex displaying the trend in the absorption.

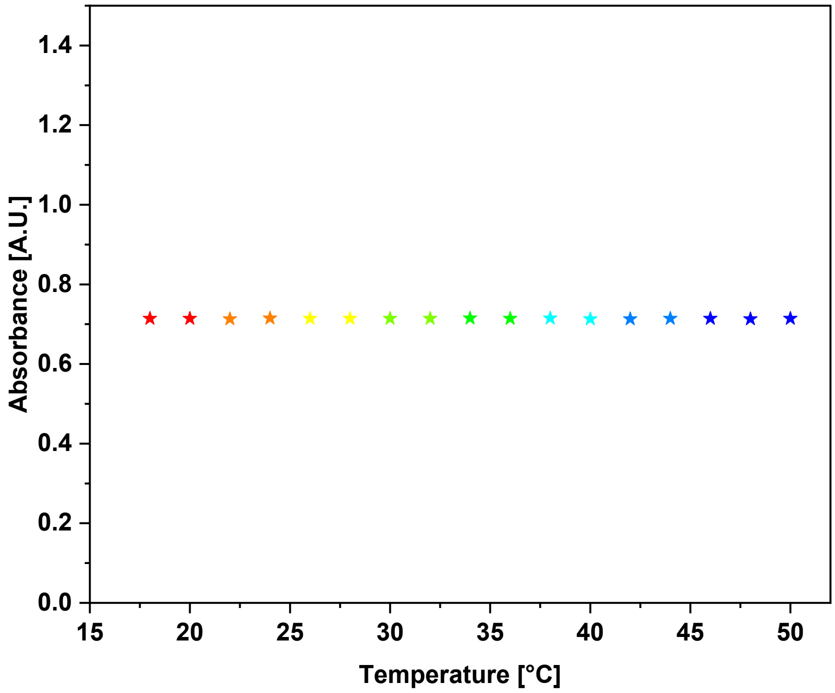

Figure S27: Temperature dependent spectra of probe 9-Cu(II) complex displaying the trend in the absorption.

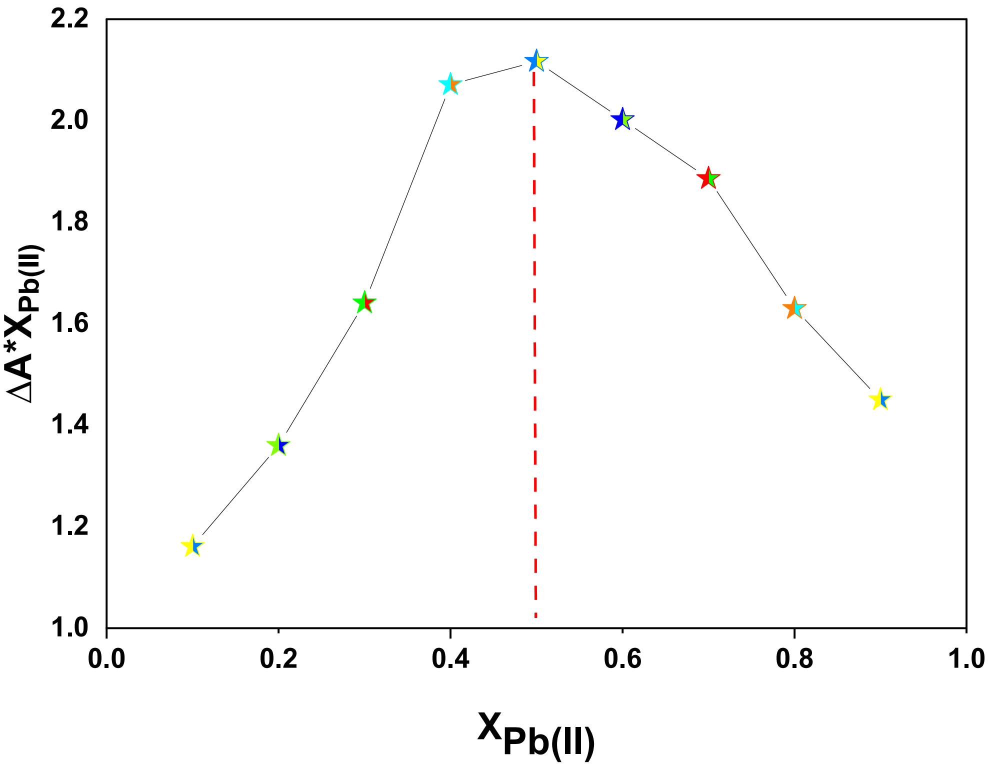

Figure S28: Job's plot analysis of probe 6 on interaction with Pb(II) ions.

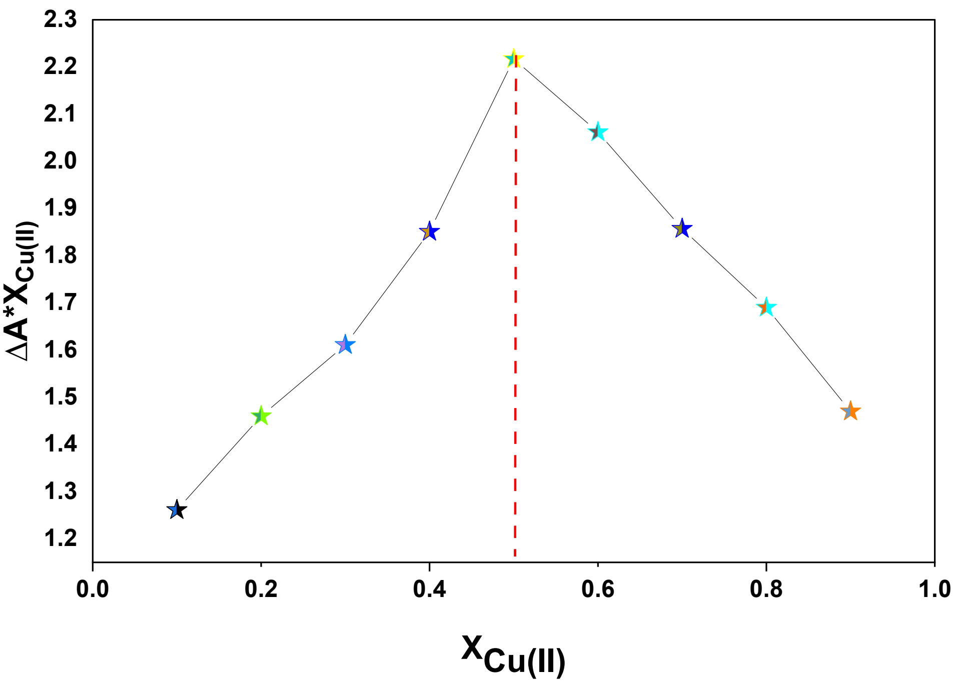

Figure S29: Job's plot analysis of probe 6 on interaction with Cu(II) ions.

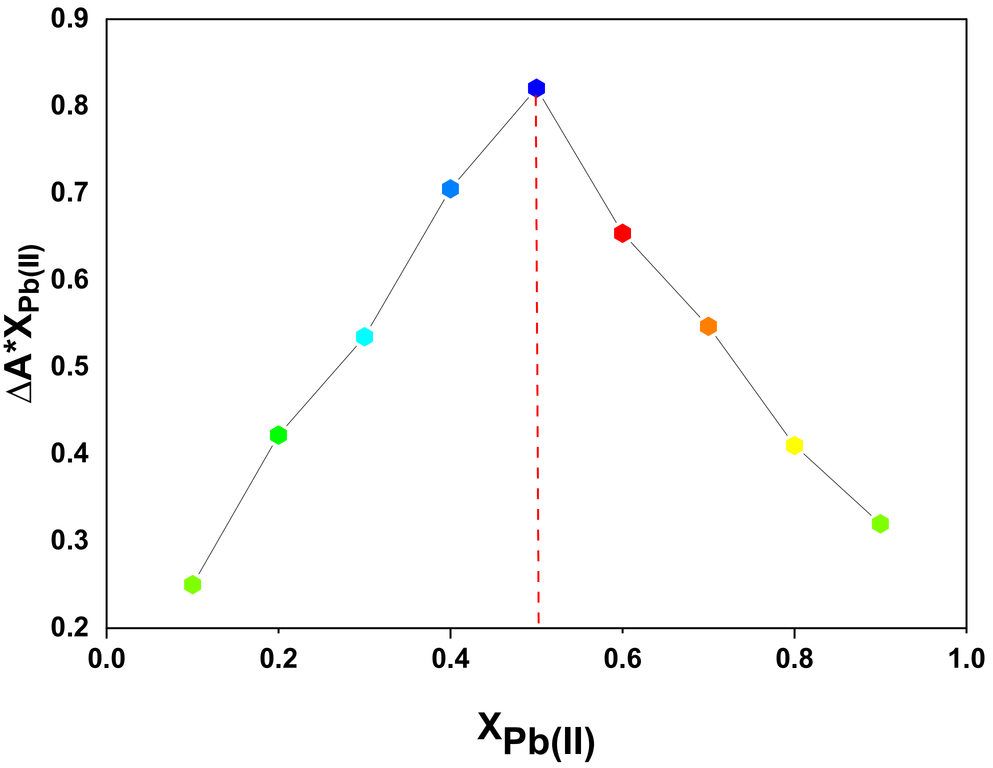

Figure S30: Job's plot analysis of probe 9 on interaction with Pb(II) ions.

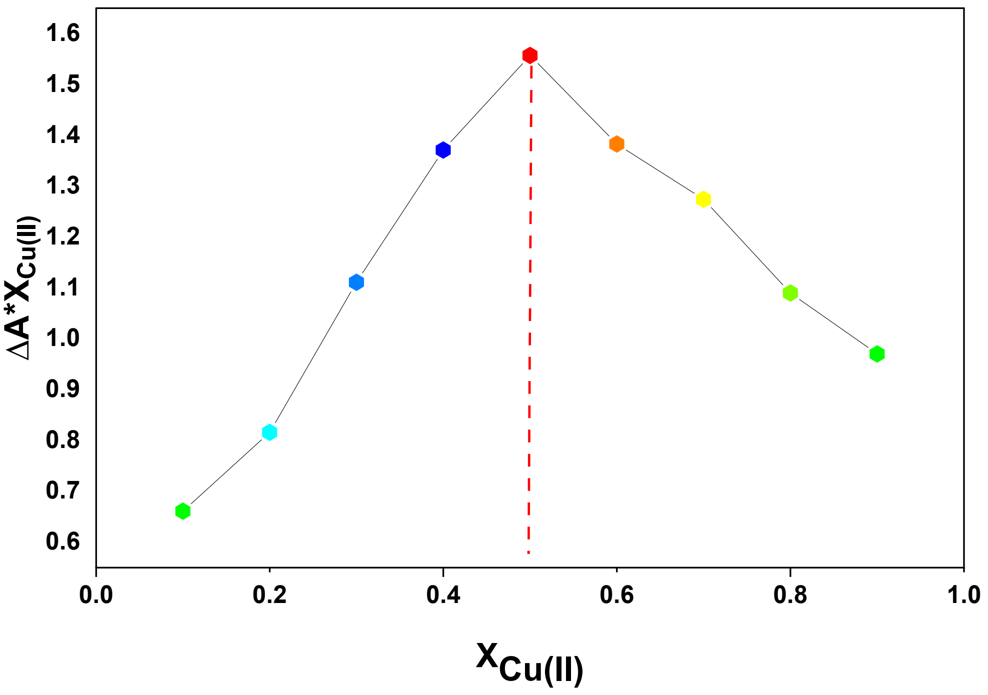

Figure S31: Job's plot analysis of probe 9 on interaction with Cu(II) ions.

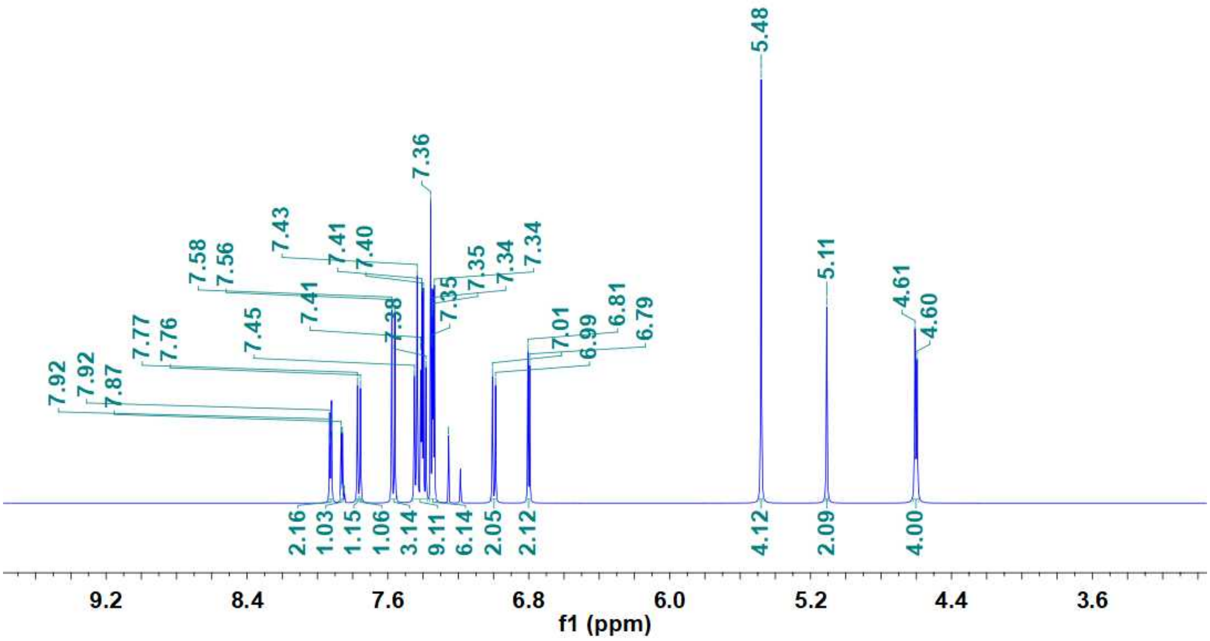

Figure S32: ^{1}H-NMR of probe 6 on interaction with the metal ion.

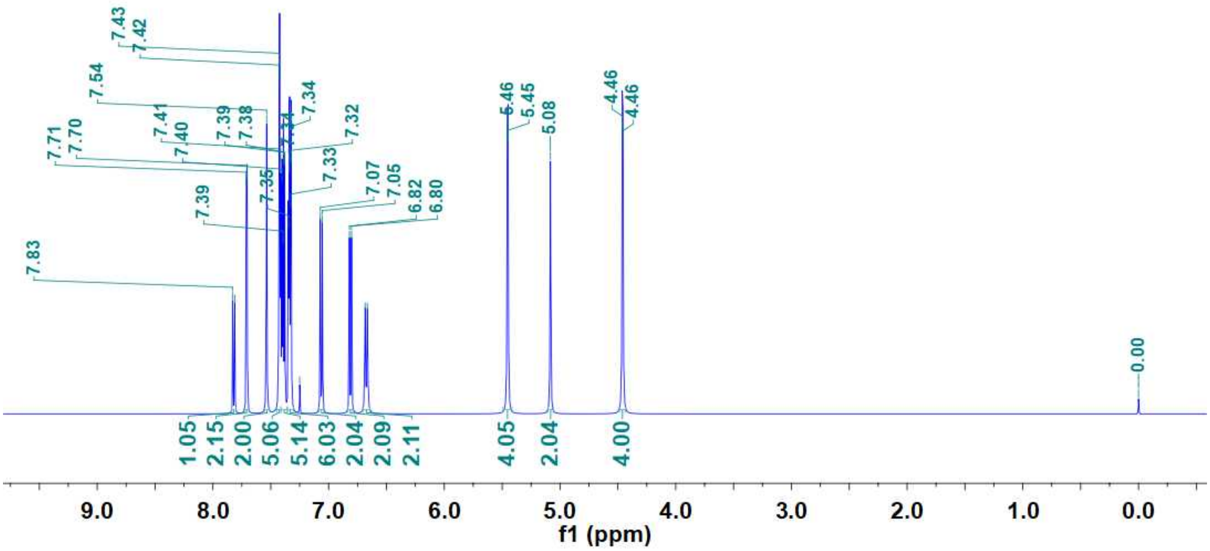

Figure S33: ^{1}H-NMR of probe 9 on interaction with the metal ion.

Symbol	X	Y	Z
C	12.60647	-1.54244	0.83657
C	11.29011	-1.46552	0.3736
C	10.84751	-0.32516	-0.31641
C	11.74121	0.734	-0.53227
C	13.06251	0.65323	-0.07726
C	13.4975	-0.48536	0.60883
H	12.93789	-2.42537	1.37401
H	10.5952	-2.27708	0.55922
H	11.40315	1.6255	-1.05369
H	13.74455	1.47959	-0.25057
H	14.52007	-0.54812	0.96727
C	9.4425	-0.25149	-0.85562
H	9.18927	0.77608	-1.14472
H	9.31923	-0.89666	-1.73744
O	8.52269	-0.71291	0.18683
C	7.16652	-0.78823	-0.09292
C	6.58189	-0.40685	-1.3081
C	6.36165	-1.2847	0.94944
C	5.19972	-0.53128	-1.46955
H	7.18063	-0.01723	-2.12165
C	4.99088	-1.40192	0.77481
H	6.84096	-1.56774	1.87918
C	4.37173	-1.02952	-0.44411
H	4.74906	-0.23716	-2.41298
H	4.38868	-1.78604	1.59106
C	2.93696	-1.13624	-0.68544
H	2.58971	-0.78972	-1.65825
C	1.98367	-1.6129	0.14951
C	0.55906	-1.61915	-0.25746
H	2.23228	-1.97261	1.14127
C	-0.41781	-2.27942	0.6549

C	0.02649	-3.35816	1.44944
C	-1.76055	-1.81701	0.82597
C	-0.7816	-3.95283	2.4154
H	1.02808	-3.74343	1.29331
C	-2.54564	-2.40246	1.8415
C	-2.07035	-3.45127	2.6267
H	-0.41139	-4.78979	2.9977
H	-3.56246	-2.05471	1.98247
H	-2.7144	-3.89368	3.38035
O	0.21037	-1.04468	-1.32258
N	-2.29789	-0.75609	0.04996
C	-2.64251	-1.03034	-1.37782
C	-3.1989	0.19406	0.73568
H	-2.82955	0.30977	1.75973
H	-4.23824	-0.16312	0.80054
H	-2.56091	-0.08728	-1.92467
H	-1.88094	-1.69405	-1.77773
C	-3.22951	1.52138	0.04444
C	-2.21394	2.43716	-0.17915
C	-4.00051	-1.63516	-1.5719
C	-5.25092	-1.03063	-1.60441
N	-2.79575	3.45577	-0.87567
N	-4.14217	3.19608	-1.08625
N	-4.38935	2.01532	-0.52635
N	-4.17361	-2.99857	-1.75546
N	-5.46532	-3.26509	-1.90465
N	-6.13545	-2.0479	-1.8126
H	-5.53529	0.00326	-1.48317
H	-1.17041	2.4185	0.0822
C	-2.21863	4.71874	-1.34821
C	-7.59915	-2.02093	-1.86958
C	-1.99834	5.73002	-0.23758

C	-3.06597	6.11693	0.59004
C	-0.73493	6.30544	-0.04216
C	-2.86742	7.06376	1.59822
H	-4.04441	5.66923	0.44281
C	-0.53719	7.25855	0.96475
H	0.09576	6.00988	-0.67763
C	-1.60276	7.63791	1.78711
H	-3.69699	7.35575	2.23423
H	0.44521	7.69732	1.10734
H	-1.45108	8.37412	2.56998
C	-8.25291	-2.00924	-0.49849
C	-7.99254	-3.04391	0.41629
C	-9.13428	-0.98052	-0.13784
C	-8.60688	-3.04461	1.67142
H	-7.30261	-3.83561	0.13963
C	-9.75284	-0.98315	1.11867
H	-9.33628	-0.17436	-0.83798
C	-9.48983	-2.01493	2.02521
H	-8.4002	-3.84759	2.37209
H	-10.43202	-0.18029	1.38758
H	-9.96705	-2.01813	3.00017
H	-7.90394	-1.14555	-2.44994
H	-7.88294	-2.91639	-2.4301
H	-1.27878	4.49789	-1.86258
H	-2.93033	5.09392	-2.08921

Table S1: Cartesian co-ordinates of probe 6

Symbol	X	Y	Z
C	-14.26535	-0.53299	-0.77069
C	-14.17929	-0.74864	-2.15008
C	-12.93176	-0.97455	-2.73389
C	-11.77345	-0.98896	-1.94943

C	-11.85465	-0.77336	-0.56991
C	-13.11005	-0.54317	0.01143
C	-8.24273	-0.87368	0.17232
C	-7.11848	-0.94704	-0.67002
C	-5.84254	-0.95953	-0.13198
C	-5.6319	-0.89806	1.26457
C	-6.77048	-0.82497	2.08454
C	-8.06465	-0.81308	1.55968
C	-4.31139	-0.90867	1.88764
C	-3.10483	-0.96292	1.28312
C	-1.85882	-0.98307	2.09074
C	-0.54142	-1.01082	1.39335
O	-1.90713	-0.98615	3.32597
C	-0.36556	-0.84927	0.00689
C	0.89469	-0.86387	-0.57637
C	2.06581	-1.06081	0.19724
C	1.8847	-1.21506	1.59536
C	0.61948	-1.18977	2.16461
N	3.32504	-1.12623	-0.39767
C	3.57982	-0.34939	-1.61958
C	4.49148	-1.38643	0.46782
C	5.71989	-1.82865	-0.2672
C	3.73018	1.12989	-1.38175
C	6.82878	-1.10293	-0.65838
N	7.64238	-2.01532	-1.24322
N	7.07764	-3.24743	-1.21237
N	5.91953	-3.13392	-0.62413
C	2.79798	2.14846	-1.43928
N	3.48101	3.26126	-1.07059
N	4.76843	2.96112	-0.79574
N	4.92022	1.67661	-0.9822
C	8.97915	-1.84008	-1.81303

C	10.05175	-1.59748	-0.76802
C	10.81596	-0.4256	-0.79448
C	11.81778	-0.20856	0.15731
C	12.05812	-1.16303	1.14713
C	11.2959	-2.33618	1.18053
C	10.30053	-2.55387	0.22753
C	3.01556	4.64873	-0.97411
C	1.76317	4.7936	-0.13405
C	0.59255	5.31762	-0.69405
C	-0.56373	5.46056	0.08018
C	-0.55786	5.07286	1.42095
C	0.60909	4.54817	1.98765
C	1.76316	4.41314	1.21627
H	-15.23013	-0.3532	-0.30508
H	-15.07682	-0.73825	-2.76151
H	-12.85454	-1.14132	-3.8046
H	-10.80626	-1.16396	-2.40605
H	-13.186	-0.36994	1.08303
H	-7.27596	-0.99553	-1.74265
H	-4.99374	-1.02118	-0.80569
H	-6.64123	-0.77935	3.16265
H	-8.91063	-0.7587	2.23392
H	-4.29743	-0.87123	2.97618
H	-3.02705	-1.01063	0.20283
H	-1.21847	-0.6996	-0.64634
H	0.95899	-0.74928	-1.65144
H	2.73639	-1.33981	2.25093
H	0.50646	-1.29826	3.23836
H	4.49873	-0.72971	-2.06674
H	2.78663	-0.53822	-2.34306
H	4.73504	-0.50379	1.07776
H	4.21911	-2.19962	1.14329

H	1.7466	2.16343	-1.67915
H	9.17147	-2.76074	-2.3703
H	8.94515	-1.01118	-2.52596
H	10.62913	0.32249	-1.56139
H	12.40369	0.70542	0.1255
H	12.83358	-0.99589	1.88897
H	11.47966	-3.08271	1.94776
H	9.70805	-3.46459	0.25661
H	2.84368	5.03375	-1.9846
H	3.85798	5.19689	-0.54417
H	0.58249	5.61706	-1.7395
H	-1.46576	5.86825	-0.36668
H	-1.45627	5.17437	2.02233
H	0.61763	4.24159	3.02929
H	2.66642	4.00205	1.65962
H	7.05891	-0.05094	-0.58595
O	-9.45299	-0.87456	-0.45845
C	-10.63783	-0.79901	0.32925
H	-10.6123	0.1063	0.9519
H	-10.68444	-1.66802	1.00241

Table S2: Cartesian co-ordinates of probe 9
